# Recent advances in wearable iontronic sensors for healthcare applications

**DOI:** 10.3389/fbioe.2023.1335188

**Published:** 2023-12-15

**Authors:** Sung-Geun Choi, Se-Hun Kang, Ju-Yong Lee, Joo-Hyeon Park, Seung-Kyun Kang

**Affiliations:** ^1^ Department of Materials Science and Engineering, Seoul National University, Seoul, Republic of Korea; ^2^ Research Institute of Advanced Materials (RIAM), Seoul National University, Seoul, Republic of Korea; ^3^ Nano Systems Institute SOFT Foundry, Seoul National University, Seoul, Republic of Korea

**Keywords:** iontronics, wearable sensor, healthcare, electrochemistry, polymer

## Abstract

Iontronic sensors have garnered significant attention as wearable sensors due to their exceptional mechanical performance and the ability to maintain electrical performance under various mechanical stimuli. Iontronic sensors can respond to stimuli like mechanical stimuli, humidity, and temperature, which has led to exploration of their potential as versatile sensors. Here, a comprehensive review of the recent researches and developments on several types of iontronic sensors (e.g., pressure, strain, humidity, temperature, and multi-modal sensors), in terms of their sensing principles, constituent materials, and their healthcare-related applications is provided. The strategies for improving the sensing performance and environmental stability of iontronic sensors through various innovative ionic materials and structural designs are reviewed. This review also provides the healthcare applications of iontronic sensors that have gained increased feasibility and broader applicability due to the improved sensing performance. Lastly, outlook section discusses the current challenges and the future direction in terms of the applicability of the iontronic sensors to the healthcare.

## 1 Introduction

Public healthcare is undergoing a revolutionary transformation with the advent of wearable sensors (Majumder et al., 2017). These sensors enable the monitoring of a wide range of human signals, including complex body movements, physiological conditions, skin temperature, and humidity (Amjadi et al., 2016). The data from these bodily signals play a crucial role in early disease detection and health monitoring (Majumder et al., 2017; Lou et al., 2020). Conventional wearable sensors primarily rely on electronic conductors. Typically, electronic conductors, such as metals or semiconductors, are known for their rigidity (Kim et al., 2021). Rigid material-based sensors are often made flexible through various strategies such as thin-film deposition and patterning, or conductive filler-polymer composite. Nonetheless, electronic pathways can be damaged or worn down under significant and repetitive mechanical stress, resulting in the degradation or loss of electrical conductivity. This highlights the need for more robust wearable sensors that can withstand mechanical stress and maintain their performance.

Iontronic sensors, in contrast to conventional electronic sensors, operate by manipulating ions in an electrolyte which can present throughout the internal sensor and transport under an electric field (Xiong et al., 2022). These characteristics make iontronic sensors exceptionally resilient in terms of their electrical performance even under various mechanical stresses because the homogeneous distribution of ions within electrolyte ensures that they do not lose conductivity under mechanical strain (Wu et al., 2021b; Dechiraju et al., 2022). Furthermore, iontronic sensors are inherently soft due to large amount of liquid phase in polymer matrix like hydrogel, organogel, and ionogel (Sun et al., 2012). Iontronic sensors are also versatile in enhancing their mechanical properties by designing intermolecular interactions within the sensor, such as high toughness, self-healing capability, or strain-stiffening behavior (Boahen et al., 2022; Wang et al., 2022b). Iontronic sensors with their exceptional mechanical characteristics, are studied as wearable sensors capable of measuring various stimuli such as pressure, strain, humidity, and temperature (Kim et al., 2021). This is because the electrical property of iontronic sensors can change in response to not only mechanical deformation but also external stimuli, such as temperature or humidity, which alters ion mobility and dissociation (Tepermeister et al., 2022). Furthermore, these diverse sensing mechanisms have propelled iontronic sensors into the realm of multi-modal sensors, capable of measuring various stimuli with a single sensor (Wen et al., 2021). This versatility makes them promising candidates for a wide range of applications, from healthcare to environmental monitoring and beyond (Dechiraju et al., 2022).

This review comprehensively explores the recent developments in wearable iontronic sensors designed for healthcare. It introduces the structure of iontronic sensors and material clusters of each component. The system comprises an ion transport layer and electrodes. It discusses materials consisting of ion transport layer and the characteristics arising from intermolecular interactions. The electrode subsection includes strategies for maintaining conductivity under large deformation of the sensor. After that, the review covers various types of iontronic sensors, including pressure, strain, humidity, temperature, and multi-modal sensor. It describes each sensing mechanism, the improvement strategies for sensing performance, and favorable applications. Especially, we deal with the detailed discussion of strategies for enhancing sensing performance. Finally, we discuss the current challenges and outlook of iontronic sensors in terms of healthcare.

## 2 Material

Iontronic sensors consist of an ion transport layer and electrodes, and they need to be soft like the skin to function as wearable sensors (Kim et al., 2021). They should also be capable of withstanding various physical stimuli like pressure, bending motion, and stretching, and maintain stability in diverse environmental conditions (Xiong et al., 2022). In this section, we will discuss recent researches on various materials used for the ion transport layer and electrodes, as well as material design strategies to enhance their mechanical and electrical performances.

### 2.1 Ion transport layer (ITL)

The ITL is a critical component of iontronic sensors, allowing them to detect external stimuli by altering electrical properties like resistance, capacitance, and impedance in response to mechanical deformation such as stretching, or bending, as well as changes in humidity or temperature (Wu et al., 2021b). This layer is composed of polymers for structural support and ions for conductivity (Park et al., 2022). Various stimuli can cause the ITL to deform or change internal temperature and humidity, resulting in variations in ionic conductivity and enabling the detection of external stimuli (Fernandes et al., 2019; Li et al., 2022a). ITLs are usually categorized based on ion transport methods within the polymer matrix. There are solvent-based ITL that ionize inorganic salts using a solvent, ionic liquid -based ITL using ionic liquid, and liquid-free ITL in solid-state forms, often containing mobile ions like polyelectrolytes (Sun et al., 2019; Qin et al., 2023). Each of these types has unique characteristics depending on the ion transport method. In brief, solvent-based ITLs have high ionic conductivity (1–10 S·m^-1^) and biocompatibility, but long-term use is challenging due to solvent evaporation (Wu et al., 2021a). Ionic liquid-based ITLs have ionic conductivity on the order of 10 mS·m^-1^ to 100 mS·m^-1^, good thermal stability, and high dry-resistance based on high boiling point and low vapor pressure of ionic liquid (Zhang et al., 2023a). However, ionic liquid-based ITLs have low biocompatibility due to the toxicity of ionic liquid. And the performance of ionic liquid-based ITLs could degrade due to liquid leakage caused by severe deformation such as squeezing (Lei and Wu, 2019). Liquid-free ITLs are highly stable in various environments and durable under mechanical deformation. However, they tend to have low ionic conductivity (1 m–10 mS·m^-1^) because the ions are bound to the polymer chain, limiting their mobility (Shi et al., 2018). Table 1 systematically organizes recent research on ITLs, categorizing studies into solvent-based ITL, ionic liquid-based ITL and liquid-free ITL for a comprehensive overview.

**TABLE 1:** Characteristics of solvent-based ITL, ionic liquid-based ITL, and liquid-free ITL.

Type	Approach	Polymer	Salt	Solvent or ionic liquid	Conductivity	Lowest working temperature	Residual weight	Conductivity reduction	References
Solvent-based ITL	hydrogel	PAAm/carrageenan	LiBr	water	12 S·m^-1^	−78.5°C	87% after 4 days (40% RH, 25°C)	-	Wu et al. (2021a)
Solvent-based ITL	organohydrogel	PAMPS/PAAm	LiCl	Water/EG	0.51 S·m^-1^	−20°C	53% after 1 day @(10% RH, 12°C)	-	Lou et al. (2019)
Solvent-based ITL	organohydrogel	PAAm/SA/TEMPO-oxidized cellulose nanofibrils	CaCl_2_	Water/DMSO	1.25 S·m^-1^ @RT	−20°C	∼90% after 3 days @(60% RH, 25°C)	-	Cheng et al. (2022)
Solvent-based ITL	organohydrogel	PVA/TEMPO-oxidized cellulose nanofibrils	NaCl	Water/DMSO	3.2 S·m^-1^	−70°C	40% after 1 day @(15% RH, 40°C)	-	Ye et al. (2020)
Solvent-based ITL	organohydrogel	PHEA/SA	CaCl_2_, KCl	Water/Gly	0.765 S·m^-1^	−80°C	90% after 10 days @(45% RH, 20°C)	-	Song et al. (2020)
Solvent-based ITL	organohydrogel	Xanthan gum/PAAm	FeCl_3_	Water/Gly	0.51 S·m^-1^	−40°C	99% after 7 days @(50% RH, 25°C)	-	Yu et al. (2021)
IL-based ITL	Ionogel	Disulfide bonds-based PU	-	[EMIM][TFSI]	11.9 mS·m^-1^	-	∼100% after 28 days	-	Yang et al. (2023a)
IL-based ITL	Ionogel	P(UA-*co*-ACMO)	LiTFSI	[EMIM][TFSI]	218 mS·m^-1^	−25°C	98.9% after 7 days @(100°C)	-	Zhang et al. (2023b)
IL-based ITL	Ionogel	PDMAPS	-	[EMIM][ESO4]	10 mS·m^-1^	−10°C	-	3% after 1 day @(60% RH, 25°C)	Lei and Wu (2019)
IL-based ITL	Ionogel	PAA	-	[EMIM][ESO4]	-		-	33.7% after 1 day @(60% RH, 25°C)	Lei and Wu (2019)
IL-based ITL	Ionogel	PAMPS grafted with METAC	-	[EMIM][TFSI]	119 mS·m^-1^	−5°C	97.5% (<170°C)	-	Li et al. (2019)
		TA							
Liquid- free ITL	Salt-in- polymer	Poly(BA-*co*-MEA)	LiTFSI	-	6.3 mS·m^-1^	-	∼100% after 75 h (@100°C)	-	Shi et al. (2018)
Liquid- free ITL	Salt-in-polymer	PEO crosslinked with 1,3,5-triformylbenzene by imine bonds (DC-PEO)	LiTFSI	-	20.4 mS·m^-1^	−35°C	∼100% after 4 months (@RT)	∼100% after 4 months (@RT)	Zhang et al. (2021)
Liquid-free ITL	Poly(ionic liquid)	Poly([EIC_6_A][TFSI]-*co*-BA)	-	-	85.8 mS·m^-1^	−20°C	∼100% after 28 days (@100°C)	-	Ming et al. (2021)
Liquid-free ITL	Poly(ionic liquid)	Poly(AA/ChCl-*co*-MA/ChCl)	-	-	43 mS·m^-1^	−20°C	-	-	Chen et al. (2020)

#### 2.1.1 Solvent-based ITL

Solvent-based ITLs use polar solvents to dissociate ions and create mobile ions within the layer. For instance, water-based hydrogels, which are polymer matrices with over 90% water content, have been a significant focus in the development of iontronics (Liu et al., 2021). This is due to their low Young’s modulus, which is similar to the skin, high ionic conductivity, and excellent biocompatibility (Dechiraju et al., 2022). The Suo group has developed highly stretchable and tough elastomeric hydrogels by utilizing strong covalent bonds between polyacrylamide polymers (PAAm) and weak ionic bonds between alginate chains and Ca^2+^ ions (Sun et al., 2012). The strong bonds remain intact during stretching, allowing only an increase in chain length, which determines the elasticity of materials, while weak bonds break during stretching, dissipating stress energy within the hydrogel, thus preventing crack propagation and increasing toughness (Gong, 2010; Chen et al., 2015). The design of the polymer network in consideration of such mechanical behavior has drawn inspiration from various molecular interactions, including ionic interaction, ion-dipole interaction, hydrogen bonding, aromatic interaction, dipole-dipole interaction, and van der Waals interaction (Park et al., 2022). However, hydrogels have inherent limitations, such as high vapor pressure leading to unavoidable evaporation issues and a high melting temperature, restricting their usable temperature range (Zhao et al., 2020).

**FIGURE 1:**
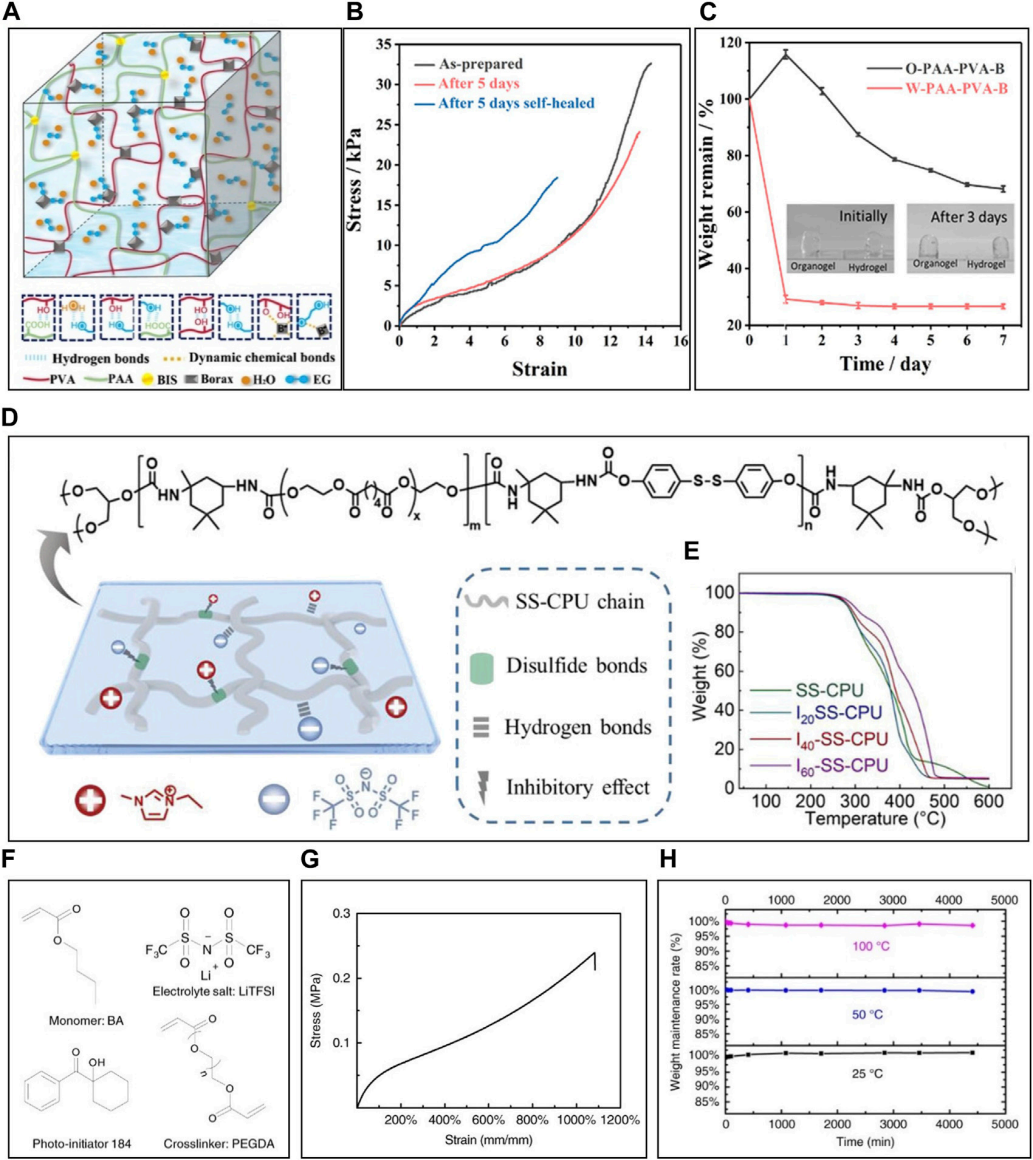
Categories and individual characteristics of ITL. **(A–C)** Solvent-based ITL, **(D,E)** IL-based ITL, and **(F–H)** liquid-free ITL. **(A)** Structure, **(B)** stretchability, and **(C)** solvent-retaining capability of solvent-based ITL. **(D)** Structure, and **(E)** thermal stability of IL-based ITL. **(F)** Structure, and **(G)** stretchability, and **(H)** weight maintenance rate of liquid-free ITL. Reprinted with permission from **(A–C)** Su et al. (2020), **(D–E)** Yang et al. (2023a), **(F–H)** Shi et al. (2018).

To overcome these fundamental limitations, some researchers have developed organohydrogels by adding hygroscopic organic solvents like ethylene glycol (EG), dimethyl sulfoxide (DMSO), glycerol (Gly) which have polar groups such as hydroxyl group (Rong et al., 2017). They are well miscible with water thanks to their hydrophilic group and break the hydrogen bonds between H_2_O molecules, prohibiting water from crystallization and enabling the ITL to operate at even subzero conditions, even though some of the solvents have high melting points (Cheng et al., 2022). Cheng et al. (2022) developed an organohydrogel by polymerizing PAAm, sodium alginate (SA), and TEMPO-oxidized nanofibrils in a Water/DMSO binary solution, followed by soaking in a CaCl_2_ solution. Increasing DMSO volume (20% - 30%) lowered the freezing and thawing peak from −15.7°C to −42.3°C, enhancing the anti-freezing effect despite the high melting point (19°C) of DMSO. This is because its increased quantity hindered water crystallization, achieving a low glass transition temperature (T_g_) of −29°C. The resulting organohydrogel exhibited excellent functionality at low temperature, demonstrating high conductivity (0.97 S·m^-1^) at −20°C. Furthermore, the organic solvents have high boiling points, or low vapor pressures, alleviating the evaporative issues of solvent-based ITL and enabling the ITL to operate for extended periods at ambient temperatures (Gao et al., 2017). Su et al. (2020) developed an organohydrogel composed of polyacrylic acid (PAA), polyvinyl alcohol (PVA), borax, EG, water and N,N′-methylene bisacrylamide (BIS) as crosslinker of PAA, causing multiple hydrogen bonds, borax-induced dynamic chemical bonds and PAA-BIS covalent bonds (Figures 1A–C). The organohydrogel can stretch over 800% due to the synergistic effect of multi-types of intermolecular interactions (Figure 1B). Moreover, the weight loss ratio of the organohydrogel and the hydrogel without, EG was 31.8% and 70%, respectively after 7 days under the 70% relative humidity environment, meaning the water-retaining capability of the organohydrogel could be enhanced thanks to the strong water/EG hydrogen bonding and EG-borax dynamic chemical bonding (Figure 1C). Some researchers have opted for organogels that offer better thermal stability and environmental stability by choosing from various organic solvents (Wang et al., 2022c). These organohydrogels have allowed for the development of ITLs that outperform hydrogels in terms of stability under various conditions (Khan et al., 2023). The material clusters for solvent-based ITL includes a variety of substances such as water, Na^+^, K^+^, Cl^−^, collagen, chitosan, sodium alginate, glycerol, sorbitol, and propylene carbonate, which constitute or are friendly to the human body, allowing solvent-based ITL to be highly biocompatible. Ling et al. (2023) used collagen and PAA as the polymer matrix, water and propan-1,3-diol as solvents, and Al^3+^ and Cl^−^ as mobile ions to develop an organohydrogel (CDPAP organohydrogel). In experiments assessing its biocompatibility through the typical CCK8 method, the proliferation of HeLa cells in the CDPAP organohydrogel extract-treated group was higher than that in the control group, indicating the cell promotion of cell proliferation by the organohydrogel extract and its biocompatibility. Furthermore, a 2-day examination of cell viability demonstrated the non-toxic nature of the developed organohydrogel as most of cells remained alive.

Solvent-based ITL presents a versatile platform for sensor design, offering a broad spectrum of tunable characteristics through a variety of selection of solvents, salts, and polymer components. This adaptability facilitates the creation of sensors with tailored properties to meet specific requirements. Also, manipulating the composition of the polymer matrix, particularly the type and quantity of solvents, provides a facile means of adjusting the T_g_ (Ye et al., 2020). This, in turn, allows for the fine-tuning of mechanical properties, such as Young’s modulus. Similarly, controlling the quantity of salt in the system offers straightforward methods for tuning electrical properties (Wu et al., 2021a). The notable conductivity of solvent-based ITL facilitates substantial signal variations, making the sensor consisting of solvent-based ITL amenable to measurement using miniaturized instruments, showcasing their potential for deployment in wearable sensor technologies. The adaptability and tunability of wearable sensors with solvent-based ITL position them as promising candidates for diverse applications, wherein their specific performance characteristics can be precisely tailored to meet the demands of various sensor designs (Song et al., 2020).

#### 2.1.2 Ionic liquid (IL) -based ITL

IL-based ITL consists of a polymer matrix and IL, closely inheriting the properties of the IL, called ionogel (Chen et al., 2018). ILs are composed of large organic cations and inorganic or organic, and their asymmetric ions interfere with the formation of regular crystal lattices, resulting in weaker ionic interactions, low melting temperature, and enabling the IL-based ITL to work at very low temperature (Nordness and Brennecke, 2020; Płotka-Wasylka et al., 2020). Also, ILs have so strong electrostatic interactions between ions that it requires a significant amount of energy to break these interactions and allows individual ions to escape into the vapor phase, resulting in the low vapor pressure of ILs and allowing IL-based ITL to operate long-term with rare solvent evaporation (Zhao et al., 2021; Luo et al., 2022c). The anion and cation within an ionic liquid can be independently selected, facilitating straightforward design for an expanded window potential. Consequently, IL-based ITL not only possess notable electrochemical stability but also encompasses a wide operational potential spectrum (Lan et al., 2020). IL-based ITL allows the design of mechanical and electrical properties through intermolecular interactions between IL and the functional groups of polymer matrix (Zhang et al., 2023b). Yang et al. (2023a) produced an IL-based ITL using 1-ethyl-3-methylimidazolium bis(trifluoromethylsulfonyl) imide ([EMIM][TFSI]) and disulfide bonds-based crosslinking polyurethane (SS-CPU) (Figure 1D). Hydrogen bonds between carbonyl and carbamate groups within SS-CPU chains were replaced by hydrogen bonds between [EMIM][TFSI] and polar groups within SS-CPU. This interaction between IL and SS-CPU chains prevented IL leakage, resulting in the maintenance of the mass of IL-based ITL constant over 28 days and enhanced thermal stability with a thermal decomposition temperature of 289.1°C (Figure 1E) (Yang et al., 2023a). Furthermore, the interaction prevented the mixture from forming crystals, resulting in a low Tg of −74.6°C, high conductivity (11.9 mS·m^-1^), and the enhancement of operable temperature (−40°C). The interaction between SS-CPU and IL reduced residual strain based on the lubricant effect of IL, enhancing the mechanical properties of SS-CPU, including a tensile strength of 1.65 MPa, maximum extensibility of 900%, and Young’s modulus of 283 kPa. Additionally, IL within the polymer matrix conferred an ionic conductivity of 1.19 × 10^−2^ S·m^-1^ to the IL-based ITL.

Ionic liquid-based ITLs are actively researched for wearable sensor applications due to high ion conductivity and high environmental and electrochemical stability (Li et al., 2019). Notably, their high conductivity and broad electrochemical window allow ITLs to withstand high potentials without electrolysis (Lan et al., 2020). This resilience to high voltages is advantageous for generating measurable currents using miniaturized instruments. Furthermore, the high thermal stability and electrochemical stability of ITLs enhance the durability and reliability of wearable sensors operating in diverse environmental conditions. The combination of these properties positions ITLs as promising candidates for sensor development, particularly in applications where robust performance and adaptability to various operating conditions are essential.

#### 2.1.3 Liquid-free ITL

In liquid-based ITL, the liquid-rich component can be lost through evaporation as well as severe mechanical deformation, such as shrinkage (Luo et al., 2023). Some researchers have aimed to develop liquid-free ITL by imparting ionic conductivity characteristics to elastomers, fundamentally solving the problem of liquid loss and enhancing the environmental stability (Ming et al., 2021; Yiming et al., 2021). First, liquid-free ITL could be approached by dissolving salt in polymer, called salt-in-polymer (Yiming et al., 2021). While solvent-based ITL relies on hydrating inorganic salts to facilitate their dispersion in the solvent, liquid-free ITL utilizes polymer chains with polar groups to dissociate and disperse inorganic salts with asymmetric structures to promote ion dissociation. Shi et al. (2018) created a liquid-free ITL based on a salt-in-polymer approach by dissolving LiTFSI powder in a monomer, butyl acrylate (BA), along with a crosslinker, polyethyleneglycol diacrylate (PEGDA), followed by polymerization (Figures 1F–H). The resulting liquid-free ITL exhibited excellent stretchability, capable of expanding by up to ∼1,100%, and demonstrated remarkable environmental stability, with negligible weight loss even after subjecting it to 4,500 min at 100°C. Ionic conductivity was relatively low at 1.27 × 10^−7^ S·cm^-1^ due to the low solubility of salt in the polymer. Another approach for liquid-free ITL involves the polymerization of ILs (PIL). PILs possess ionic conductivity based on the presence of numerous ionizable groups within the polymer chains, enabling the mobility of ions. Ming et al. synthesized ionoelastomers based on PIL by copolymerizing BA with [1-(6-acryloyl-oxyhexyl)-3-ethylimidazolium][TFSI] (Ming et al., 2021). This ionoelastomer exhibits a soft Young’s modulus of 72 kPa, exceptional stretching capability with a limiting strain of 1,460%, and ionic conductivity of 8.6 × 10^−5^ S·cm^-2^. Many studies have been conducted to lower the T_g_ of liquid-free ITL to enhance their conductivity. As the T_g_ of ITL decreases, the segmental motion of polymer chains and the mobility of the polymer chains increase, leading to improved movement of mobile ions within the polymer chains and an increase in ionic conductivity. Zhang et al. (2021) developed a liquid-free ITL by crosslinking amino-terminated poly(ethylene glycol) with benzene-1,3,5-tricarbaldehyde (DC-PEO) and adding LiTFSI (DC-PEO/LiTFSI). DC-PEO/LiTFSI, with its amorphous structure expanded due to dynamic crosslinking, had a lower T_g_ of −40°C and achieved a high ionic conductivity of 20.4 mS·m^-1^, while linear-PEO/LiTFSI exhibited a higher T_g_ of −33°C due to the semi-crystalline structure of linear-PEO, resulting in low conductivity (0.242 mS·m^-1^). Furthermore, the lower T_g_ allows the liquid-free ITL to maintain an amorphous region even at lower temperatures, enabling it to retain ionic conductivity at lower temperatures. With a T_g_ of −40°C, DC-PEO exhibited measurable ionic conductivity (0.00473 mS·m^-1^) even at −35°C.

Liquid-free ITL is typically prepared using toxic inorganic salts or IL, resulting in decreasing biocompatibility. However, recent advancements have led to the development of non-toxic inorganic salts and ILs, paving the way for the creation of biocompatible liquid-free ITLs. Niu et al. (2023) successfully developed an ITL using inositol hexakisphosphate (IP6), an FDA-approved ionizable compound, and PVA (IP6-PVA ITL). The hydroxyl groups in PVA enable IP6 to form mobile protons, resulting in a liquid-free ITL with a conductivity of 33 mS·m^-1^. Experiments involving the application of IP6-IPA ITL on mice skin for 3 days and oral gavage for 42 days demonstrated excellent biocompatibility. No skin irritation or pathological signs were observed in both the skin and internal organs, confirming the outstanding biocompatibility of IP6-IPA ITL. Despite these promising developments, the quest for biocompatible liquid-free ITLs requires further research, as the field is still in the process of developing highly performing ionic liquids and inorganic salts with enhanced biocompatibility.

Liquid-free ITLs are manufactured based on thermally and electrochemically stable inorganic salts or ionic liquids, endowing sensors produced from liquid-free ITLs with exceptional thermal and electrochemical stability (Pei et al., 2022). Additionally, the absence of a liquid state within the ITLs eliminates the risk of liquid leakage (Ming et al., 2021). Consequently, performance degradation due to substantial or repetitive mechanical stimuli is significantly reduced compared to the other two types of ITLs. This is a crucial factor for wearable sensors continually exposed to various scales of mechanical stimuli, highlighting the potential for the ITL to become a robust candidate for future wearable sensors (Kim et al., 2019). However, it's worth noting that liquid-free ITL currently exhibits lower conductivity compared to the other two types of ITLs. Moreover, while the other two types of ITLs can easily design mechanical and electrical properties by adjusting the amount of liquid state within the ITL to control the T_g_, liquid-free ITLs is hard to adjust T_g_, making the design of material properties relatively more complex. For these reasons, more extensive research is needed, particularly in the development methods, to further understand and enhance the properties of liquid-free ITLs.

### 2.2 Electrode

As skin-interfaced electronics have emerged for healthcare monitoring, there is a demand for flexible and stretchable electrodes that can operate on the curved and stretching skin. In response, research has focused on developing flexible and stretchable electrodes by blending conductive materials like carbon nanomaterials (Park et al., 2022) or metal nanomaterials (nanoparticles, nanowires, and nanosheets) (Lee et al., 2012; Lee et al., 2022) with flexible and stretchable polymers. Conductive polymers like Poly(aniline) (PANI) and poly(3,4-ethylenedioxythiophene):poly(styrenesulfonate) (PEDOT:PSS) have also been used for this purpose (Fan et al., 2015; Meng et al., 2017). However, iontronic sensors measure differences in ionic conductivity based on external stimuli, which can be affected by electrode resistance (Tepermeister et al., 2022). For accurately measuring ionic conductivity with maintained conductance during mechanical deformation, it is essential to develop both highly conductive and strain-insensitive electrodes. Various research efforts have been developed to maintain a conductive pathway in mechanical deformation by introducing additional materials or structural designs.

#### 2.2.1 Electrode with multi-layered conductive layer

**FIGURE 2:**
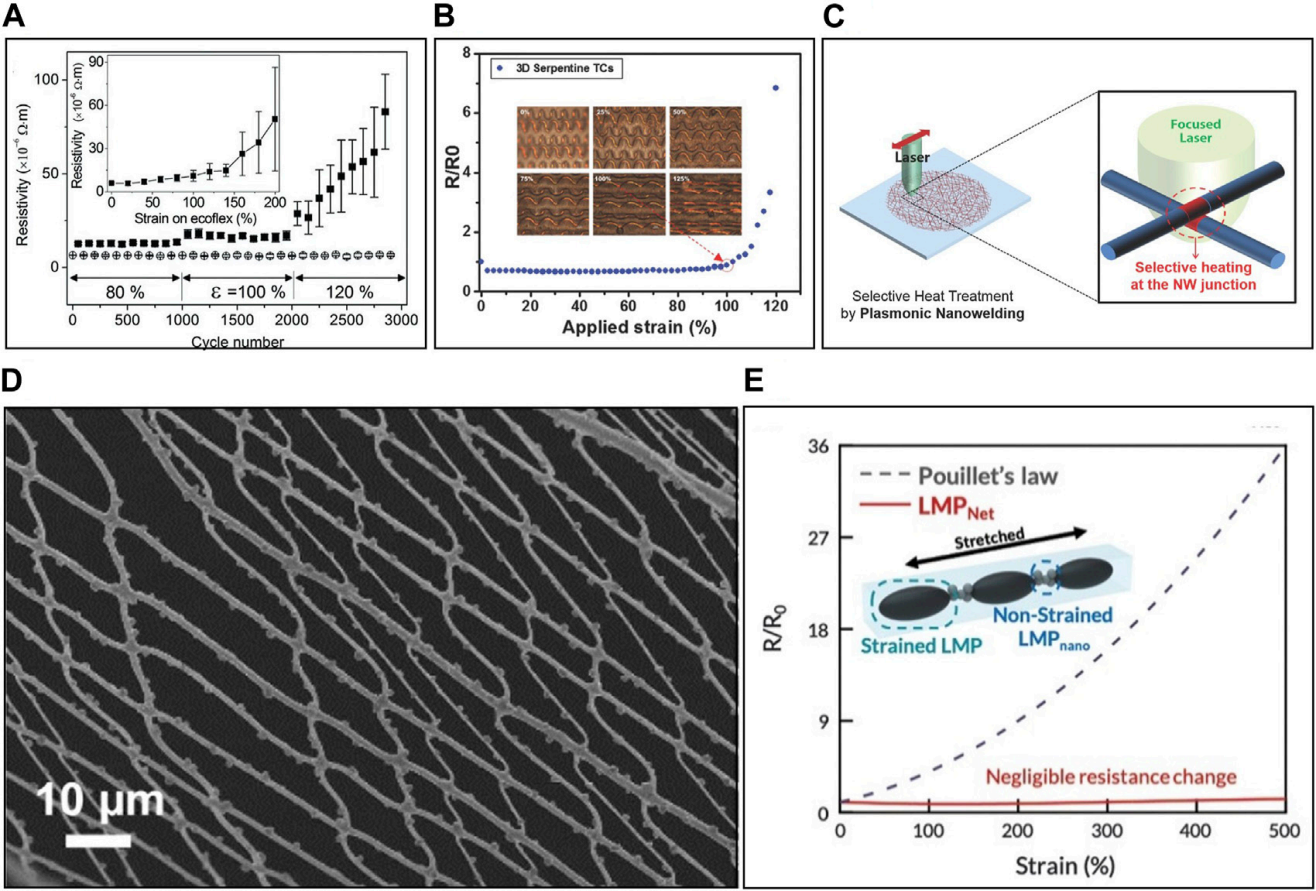
Strain-insensitive electrodes. **(A)** Resistivity changes of multilayer Au nanosheet-based on ecoflex. **(B)** Resistance changes of serpentine-structured Au within PDMS at strain. Insets show images of serpentine-structured Au at various strains. **(C)** Schematic illustration of plasmonic nanowelded Cu nanowires on PDMS. **(D)** SEM image of welded Ag grid nanopatterned electrode with external strain. **(E)** Resistance change of interconnected liquid metal based electrode at strain. Reprinted with permission from **(A)** Moon et al. (2013), **(B)** Jang et al. (2018), **(C)** Han et al. (2014), **(D)** Bai et al. (2017), **(E)** Lee et al. (2022).

Conductance decreases in mechanical deformation as the conductive fillers within the polymer matrix to separate, leading to pathway interruptions and fillers failure. Researchers have developed multi-layered conductive fillers to create sufficient conductive pathways, allowing them to be maintained up to a certain level of strain. Moon et al. (2013) created a multi-layered Au nanosheet-based electrode through several transfers of floated Au nanosheets on a water surface onto substrates. It exhibited up to eight transfers and the resulting electrode showcased a conductance change of less than two times even at 100% strain (Figure 2A). In another approach, Kim et al. (2023) developed a strain-insensitive electrode using graphene multi-layers through bar coating. Thicker graphene layers exhibited higher strain-insensitivity. Up to a certain strain, conductance maintains with sufficient conductive pathways. However, beyond this point, conductance rapidly decreases due to mechanical failure occurring in conductive fillers. To address this issue, Liu et al. (2017) introduced scroll-like graphene between graphene multi-layers, creating an electrode with a 1.54-fold resistance increase at 100% strain. The larger modulus of the scroll-like graphene prevented failure and allowed sliding along the strain direction, bridging broken graphene layers to maintain the conductive pathway. While more conductive fillers make the electrode more strain-insensitive, they reduce flexibility and increase the Young’s modulus of the electrode, leading to mechanical mismatches with the skin and ionic gel. Consequently, research is ongoing to achieve strain insensitivity without increasing the amount of conductive fillers.

#### 2.2.2 Electrode with wavy structured conductive layer

To prevent the failure of conductive layers with low toughness, some studies have developed strain-insensitive electrodes with a serpentine pattern or wavy structures to make the conductive pathway (Zhang et al., 2014; Rahimi et al., 2017). These patterns help maintain the conductive pathway and minimize changes in conductance. Jang et al. (2018). Produced an electrode by embedding a serpentine-structured Au within a Polydimethylsiloxane (PDMS) matrix. They optimized the height and width of the Au conductive line to calculate the maximum strain on Au based on external strain, finding the most strain-insensitive electrode. An electrode embedded with 6 μm height and 40 nm width Au line maintained its conductance even at 100% strain (Figure 2B) (Jang et al., 2018). In addition to serpentine patterning using lithography, wavy structures are fabricated by pre-strain. PDMS undergoes additional crosslinking through treatments such as Ar plasma or UV/O_3_ plasma, which increase their Young’s modulus like glass (Fu et al., 2010; Hanske et al., 2014). When a pre-stretched elastomer with plasma-treated glass-like surface layers is relaxed, it experiences compressive stress due to the elastomer’s strong restorative force, causing it to bend and form a wrinkled structure. The conductive layer deposited on the surface also becomes wrinkled, thus maintaining the conductive pathway even under external strain. He et al. (2022) created a strain-insensitive electrode with a graphene layer exhibiting a wrinkled structure with above method. An electrode produced under 50% pre-strain maintained conductance even at 80% strain, outperforming the electrode made from the same material without pre-strain. However, wavy structures are created through pre-stretching in one direction, making them insensitive to strain only in that direction. For practical skin applications, electrodes need to be insensitive to strain in all directions.

#### 2.2.3 Electrode with fixed conductive pathway

Compared to failure of isotropic 2D nanosheet under external strain, 1D nanowires are anisotropic, which align along the strain direction before failure occurs. Therefore, if the interconnection between nanowires is maintained, the conductive pathway remains relatively stable with the alignment of nanowires, resulting in minimal changes in conductivity (Bai et al., 2017; Ho et al., 2018). Han et al. (2014) coated Cu nanowires on flexible substrates and used plasmonic nano-welding through laser irradiation to transmit a significant amount of energy only to the interconnection points without damaging the substrate (Figure 2C). The welded points were resistant to failure from mechanical deformation, and the electrode showed minimal changes in conductance even at 250% strain. Bai et al. (2017) created a welded Ag nanopatterned electrode by forming a grid nanopattern through dry spinning of precursor and sintering. Interconnected Ag formed strong chemical bonds through sintering, which established a fixed conductive pathway that remained stable even under external strain (Figure 2D). The sheet resistance increased by only 12% at 30% strain. However, it is important to note that the strain range where nanowires only undergo alignment is relatively narrow, typically less than 100%. Beyond this point, failures occur, and conductance rapidly increases. To address this issue, research is being conducted using intrinsically stretchable materials.

#### 2.2.4 Electrode with soft conductive materials

Conductive polymers, such as polyaniline, polypyrrole, and PEDOT, have been employed to create soft electrodes with their electrical conductivity and mechanical properties (Lee et al., 2016; Lu et al., 2019; Inoue et al., 2020). Conductive polymers exhibit higher toughness compared to metals or carbon nanomaterials, making them resistant to failure by external strain. However, conductive polymers embedded within a conductive hydrogel through simple mixing can undergo reorientation, leading to changes in the conductive pathway. To address this, Lee et al. (2016) formed densely packed PEDOT:PSS pathways within a PEDOT:PSS hydrogel using freeze-drying and maintained them by replacing the solvent with, EG. The established conductive pathway remained stable under external strain without deformation or failure, showing minimal changes in conductance even at 100% strain.

Ongoing research explores stretchable electrodes using liquid metal (LM) with metallic conductivity that can endure substantial deformations at room temperature (Chiechi et al., 2008; Dickey, 2017). Yet, like conductive polymers, external strain can alter connections among LM fillers, increasing resistance. To address it, Lee et al. (2022) mixed LM into a polyurethane matrix and employed an acoustic field to create rigid LM nanoparticles in the connection points, forming a strain-insensitive electrode (Figure 2E). The connections stay stable, ensuring a consistent conductive pathway. These electrodes displayed strain insensitivity, with only a 1.41-fold increase in resistance even at 600% strain.

## 3 Iontronic pressure sensor (IPS)

### 3.1 Principle of capacitive IPS: contact area change-induced capacitive change

**FIGURE 3:**
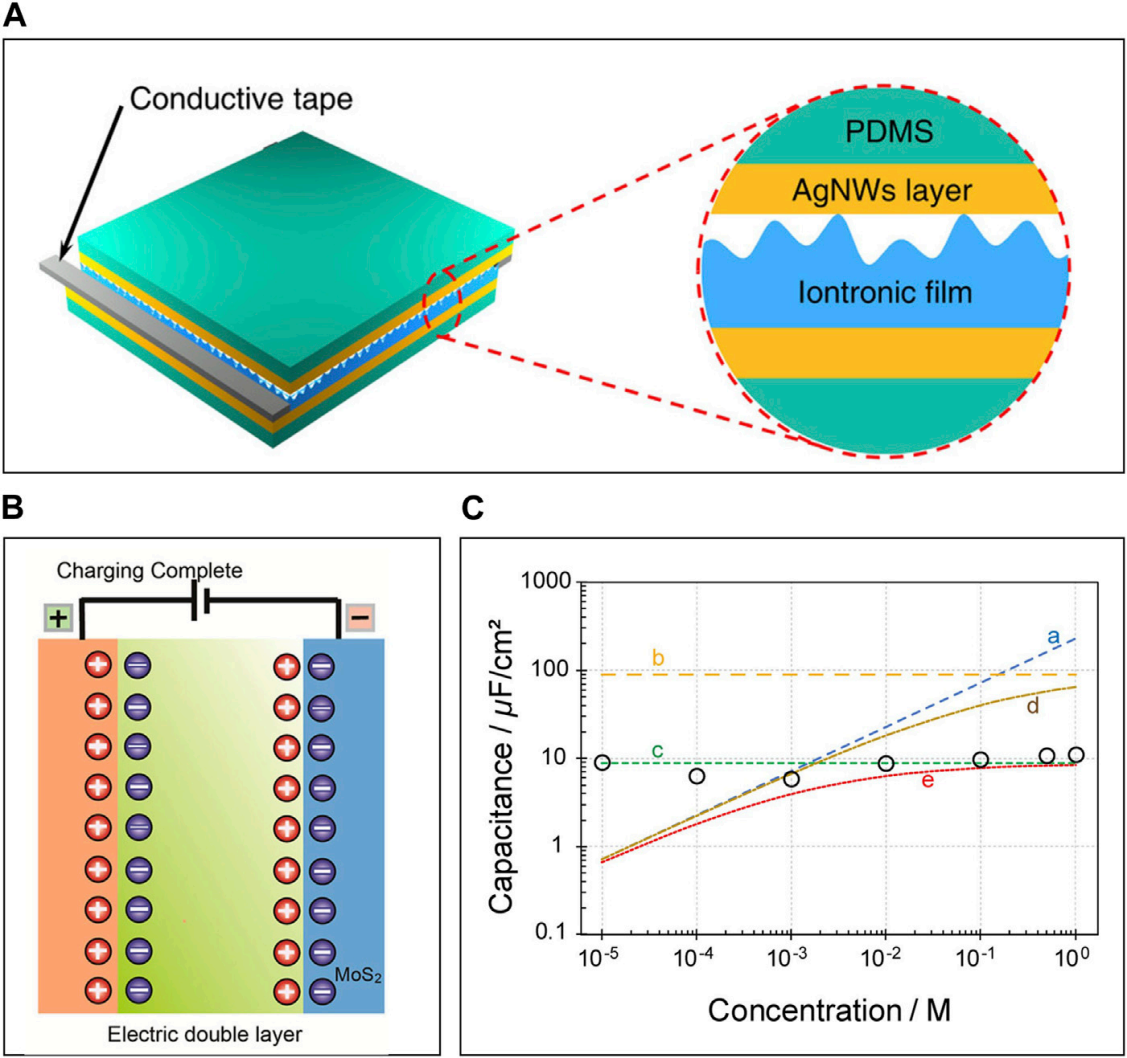
The principle of IPS. **(A)** The structure of IPS, **(B)** The formation of EDL between electrode and electrolyte (ITL) **(C)** Capacitance *versus* NaCl concentration from experiment (open circles). Reprinted with permission from **(A)** Chhetry et al. (2018), **(B)** Yuan et al. (2015), **(C)** Khademi and Barz (2020).

Pressure is a valuable signal offering insights into various health aspects, including heart rate, blood pressure and human body motion like locomotion (Gong et al., 2014; Wang et al., 2018; Meng et al., 2022). Wearable pressure sensors with softness, stretchability, and toughness are useful for effectively measuring biological pressure (Ray et al., 2019; He et al., 2020). IPSs are gaining significant attention due to their remarkable mechanical behaviors. Many IPSs adopt a sandwich configuration consisting of two electrodes separated by an ITL (Figure 3A) (Chhetry et al., 2018). When voltage is applied to the electrode, charged electrodes attract counter-ions and repel co-ions, resulting in the formation of the electrical double layer (EDL) at the electrode/electrolyte interface with a thickness of ∼1 nm (Figure 3B) (Yuan et al., 2015). The absence of the charge-transfer reaction in this process causes charge to be stored over this thin layer, giving rise to huge capacitance, called EDL capacitance (C_EDL_) that is about 100 times larger (ranging from nF·cm^-2^ to μF·cm^-2^) than that of traditional solid dielectric methods (∼pF·cm^-2^) (Khademi and Barz, 2020). This C_EDL_ is proportional to the contact area between the electrodes and ITL (Xiong et al., 2022). External pressure-induced deformations lead to alterations in the contact area between the ITL and the electrode, causing large variations in C_EDL_. This sensing mechanism enables the miniaturization of IPSs, making them portable and wearable, because their high capacitance is measurable though the small size of impedance analyzers without electromagnetic shielding.

### 3.2 Performance enhancement of IPS

In the early stages, the IPSs often employed structures resembling droplets or thin beams (Nie et al., 2012; Yang et al., 2018). These structures deform in response to pressure variations, resulting in fluctuations in the contact area between the electrode and ITL, and C_EDL_. However, achieving linear geometric changes in response to pressure with a singular structure is challenging (Chang et al., 2021). Additionally, the restricted contact surface area inherently limited sensor sensitivity (Xiong et al., 2022). To address these issues, improvements in the microstructure of ITL and hybrid capacitive electrodes were introduced (Bai et al., 2020; Gao et al., 2021; Ren et al., 2021). The capacitive pressure sensor section (the upper section) of Table 2 summarizes the recent IPSs modified through the mentioned approaches. These advancements not only elevated sensor performances but also expanded their applications in healthcare monitoring, enabling IPSs to monitor pulse waves or movements like locomotion (Xu et al., 2021; Wang et al., 2022a).

**TABLE 2:** Sensing performances of iontronic mechanical sensors in accordance with enhancement strategies.

Sensor type	Strategy	Materials	Elastic modulus	Sensitivity	Sensing range	Cyclability	References
Capacitive pressure sensor	Internal porous structure engineering	PU [BMIM][BF_4_]	3.4 kPa	9,280 kPa^-1^	2 kPa–114 kPa	5,000	Liu et al. (2022)
		Poly([VBIM][BF_4_]) crosslinked with MBA	4 kPa–133 kPa	0.002–0.02 kPa^-1^	10 Pa–1 MPa	1,000	Zhang et al. (2022c)
		[BMIM][BF_4_]					
		P(DMA-co-HEA-co-[VIM][DCA]) [HMIM[DCA]	10 kPa - 10 Mpa	1.62 nF kPa^-1^	300 Pa - 2.5 Mpa	10,000	Ren et al. (2021)
	Surface microstructure engineering	PVA	∼2.5 MPa	3,302.9 kPa^-1^	0.08 Pa - 360 kPa	5,000	Bai et al. (2022)
		H_3_PO_4_					
		PVDF-HFP [EMIM][TFSI]	750 kPa	33.7 kPa^-1^	0.36 Pa - 1.7 MPa	4,500	Yang et al. (2023b)
	Hybrid capacitance	Ti_3_C_2_T_x_ electrode	-	46,730 kPa^-1^	20 Pa - 1.4 MPa	10,000	Gao et al. (2021)
		PVA					
		KOH					
		Ti_3_C_2_T_x_ coated Au electrode	20 kPa	36,000 kPa^-1^	0.015 Pa–300 kPa	5,000	Luo et al. (2022b)
		PVA [EMIM][OTf]					
Resistive strain sensor	Polymer network modulation	PAAm/CMC	13.55 kPa	1.42 (@300%–450%)	0%–450%	500 (@ 200%)	Bai et al. (2021)
		Water					
		CaCl_2_					
		CS/HA	-	4.42 (@450%–800%)	0%–800%	300 (@ 50%)	Gao et al. (2023)
		Water					
		AlCl_3_					
	Geometry of ISS	PVA	42–175 kPa	9.21 (@ 100%)	0%–310%	-	Zhao et al. (2023a)
		Water/Gly					
		FeCl_3_					
	Microstructure engineering	POSS/TMB	0.51–1.07 MPa	7.03 (@ 100%)	0%–100%	220 (@ 100%)	Ou et al. (2022)
		LiMTFSI					
		Nanomesh PU	1.8 MPa	66.8 (@630%)	0%–630%	2,500 (200%)	Wang et al. (2022b)
		P(AAm-*co*-AA)/HA					
		CaCl_2_					
	Double conductive pathway	PAAm	0.15 kPa	13.14	0%–1,100%	100 (@ 50%)	Wang et al. (2021)
		Water					
		SC rGO					
		PAAm	295 kPa	15.47 (@240%–400%)	0%–400%	-	Dong et al. (2023)
		ChCl					
		Ga/ln					
		Ti_3_C_2_T_x_					

#### 3.2.1 Internal porous structure engineering

**FIGURE 4:**
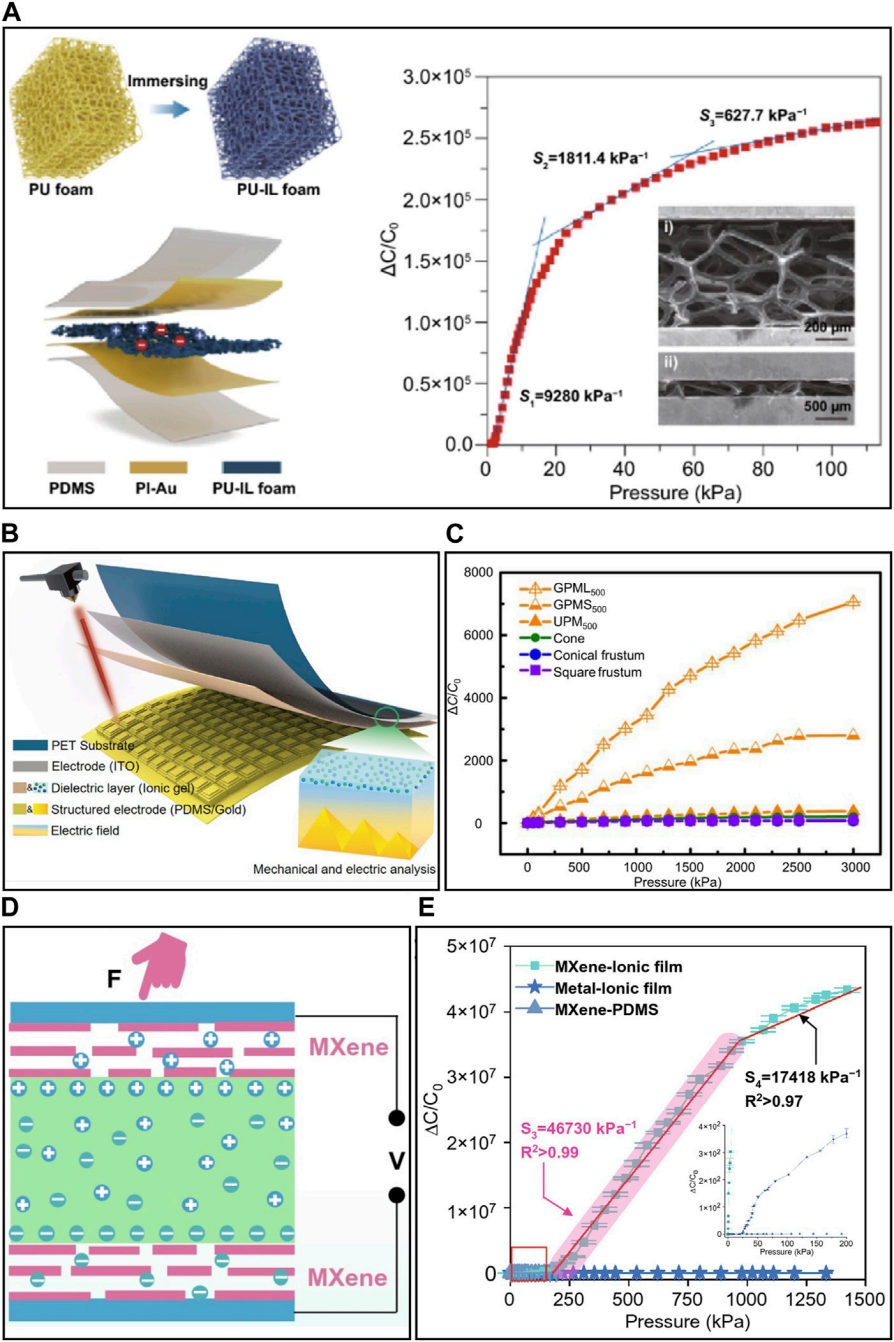
The strategies for the enhancement of sensing performance of IPS. **(A)** Internal porous structure engineering (Uniformly porous structure) and sensitivity. **(B)** Surface microstructure engineering (gradient micro-pyramidal surface) and **(C)** sensitivity. **(D)** Hybrid capacitance and **(E)** sensitivity. Reprinted with permission from **(A)** Liu et al. (2022), **(B,C)** Yang et al. (2023b), **(D,E)** Gao et al. (2021).

Many studies are currently underway to enhance sensor performance by using porous matrices, which exhibit high pressure sensitivity due to their significant compressibility. Porous matrices are often created through methods such as electrospinning nanofibers or by adjusting crosslinking to form a foam-like structure (Kwon et al., 2021; Fan et al., 2023). Due to the inherent characteristics of porous matrices, they also have a low Young’s modulus, which further contributes to their high sensitivity. Liu et al. (2022) created a 3D porous skeleton by infusing gas into polyurethane and then absorbing it with an IL during curing (Figure 4A). This process produced a matrix with 95.4% porosity and a notably low Young’s modulus of 3.4 kPa. Based on these characteristics, they achieved a high sensitivity of 9,280 kPa⁻^1^, a fine pressure resolution of 0.125%, and could even measure minute pressure of 5.2 Pa. However, it possesses a limited working range because the contact area saturates quickly due to their structural attributes. To expand the working range, research has been conducted to create multi-layer structures using materials with different Young’s modulus. Initially, soft materials make contact, and as the pressure increases, hard materials come into contact, extending the working range. Zhang et al. (2022c) developed three multi-layers with varying porosities achieved by differentiating crosslinking when polymerizing an IL. This multi-layered ITL has each responsive pressure range corresponding to their Young’s modulus, and it can measure the wide range of pressures from 10 Pa to 1 MPa. Ren et al. (2021) reported that a continuously gradient porosity of ITL widen their sensible pressure range. The gradient porosity in ITL was fabricated by inducing an electric field and generating gradient crosslinker concentrations during photopolymerization. Using this method, the pressure can measure a wide range of pressures from 300 Pa to 2.5 MPa.

#### 3.2.2 Surface microstructure engineering

Engineering the surface microstructures of ITL with 3D microstructures, such as micro-pyramids, micro-domes, and micropillars, has been studied (Cho et al., 2017; Xiong et al., 2022). This surface microstructure engineering aims to minimize the initial contact area due to gaps, resulting in low initial capacitance. This can be leveraged to amplify capacitance changes in response to pressure, ultimately achieving a wide pressure range. The initial surface microstructures undergo changes in shape and an increase in the contact area. However, at high pressures, the difficulty in further shape transformation led to lower sensitivity (Yang et al., 2023b). Bai et al. (2022) proposed a graded intrafillable architecture (GIA) by using sandpaper as a mold to tune the ionic gel surface. This architecture combines unstable microstructures with surface undercuts and grooves, which fills the internal undercut parts under pressure. This introduces a gradient based on the height of the protruding microstructure, minimizing the initial contact area. As a result, they achieved a sensitivity of approximately 3,302.9 kPa^-1^ at lower pressure (<10 kPa). In the pressure range of 10 kPa–100 kPa, the protruding structures buckle down to minimize contact area, forming more fine-scale EDL, and the device maintains a high sensitivity of 671.7 kPa^-1^. At higher pressures (>100 kPa), the GIA further enhances compressibility by filling the fine protruding parts into surface undercuts and grooves. Consequently, the sensor provides a sensitivity of 229.9 kPa^-1^ up to pressure of 360 kPa. By incorporating unstable protrusions with height gradients and surface undercuts to accommodate them, a sensor with high sensitivity for a wide pressure range was developed. However, due to the random surface, the linearity of sensitivity may be somewhat reduced. For achieving high linearity over a wide range, Yang et al. (2023b) employed CO_2_ laser technology to create programmably compressible electrode pyramids with varying heights and an ultra-thin ionic layer (Figures 4B, C). Under pressure, the ionic layer bends and sequentially contacts the programmable height electrode pyramids. This design resulted in a sensor with a sensitivity of 33.7 kPa^-1^ over a range of 1.7 MPa and high linearity of 0.99. However, the contact area between the electrode and the ITL is determined by the surface area of the electrode, limiting the maximum capacitance and sensitivity achievable.

#### 3.2.3 Hybrid capacitance

A hybrid capacitance, which combines C_EDL_ and pseudo-capacitance (C_p_), can enhance the functionality of the IPS, as it offers capacitance values significantly larger than that of C_EDL_ alone (Fleischmann et al., 2020). C_p_ arises when the ITL contacts the electrode under pressure, triggering an electrochemical reaction at the electrode. During this reaction, ions are adsorbed and move towards the electrode, resulting in charge storage. This electrochemical charge storage mechanism offers a capacitance greater than C_EDL_ (Wang et al., 2015). Gao et al. (2021) used Ti_3_C_2_T_x_ as an MXene electrode that operates based on intercalation-based C_p_ (Figures 4D, E). They combined it with PVA-KOH and achieved a high sensitivity of 46,730 kPa^-1^ in the range of 200–800 kPa and a sensitivity of 17,148 kPa^-1^ up to 1.4 MPa, demonstrating a wide working range. They also developed a high-performance device with a limit of detection sensitivity of 216.4 kPa^-1^ at 20 Pa units, owing to the rapid response of this C_p_. Going beyond simple C_p_, MXene electrodes were fabricated into a 3D microstructure to achieve even higher sensitivity. Luo et al. (2022b) coated MXene onto a substrate with a slant hierarchical structure, created by sputtering Au onto a 3D-molded surface. Using these microelectrodes as a basis, they developed a pressure sensor with a high sensitivity of 36,000 kPa^-1^.

### 3.3 Application of IPS

**FIGURE 5:**
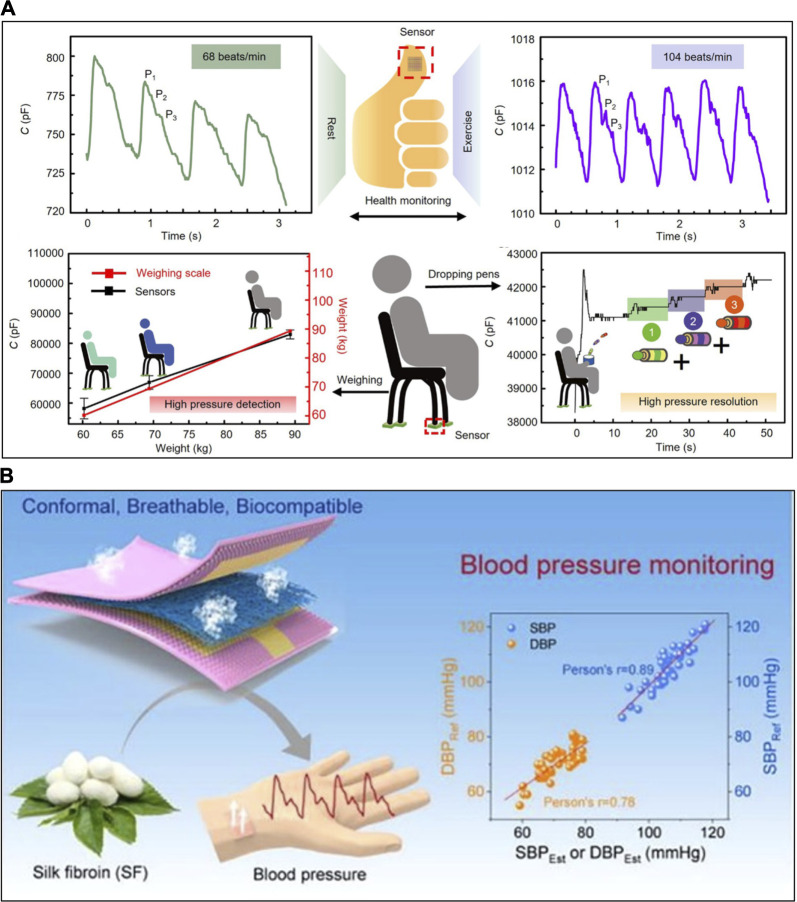
The application of IPS. **(A)** Monitoring of fingertip pulse waves and high-precision scaling with high-pressure resolution by the gradient micro-pyramidal surface structure. **(B)** Blood pressure monitoring by silk nanofibrous IPS. Reprinted with permission from **(A)** Yang et al. (2023b), **(B)** Wang et al. (2023b).

Based on these improved sensing capabilities, IPS has advanced beyond slow and large motions, such as locomotion, expanding its applications to various real-life and healthcare scenarios. Yang et al. (2023b) demonstrated that IPS with gradient micro-pyramidal surface can recognize tiny pressure even under high-pressure conditions, surpassing the performance of commercial weight scales (Figure 5A). The IPS with high linearity was validated for its ability to sense linearly high pressures corresponding to 60.2, 69.4, and 89.3 kg, comparable to commercial weight scales. Furthermore, they confirmed its exceptional performance by successfully detecting subtle pressures of 5.8 g of a pen (∼145 Pa), even when subjected to high pressure from a human weighing 69.4 kg (∼2000 kPa). Also, this outstanding capability to detect fine pressure enables the monitoring of subtle pulse waves originating from the fingertip. It can not only measure heart rate but also recognize various components of the pulse waves, including percussion, tidal waves, and dicrotic waves.

To achieve long-term blood pressure monitoring with adherence to the skin, it is crucial for the sensor to conform to the skin’s surface and move simultaneously, ensuring the accurate reception of pressure signals. However, skin secretion such as sweat and lipid can hinder this conformal contact, making it necessary to address this issue at the skin/sensor interface. Wang et al. (2023b) developed an electrospun silk nanofibrous IPS designed to facilitate the evaporation of sweat (Figure 5B). As an ITL, they produced a deep eutectic solution using choline chloride as the hydrogen bond acceptor and Gly as the hydrogen bond donor. This nanofibrous IPS demonstrated a gas permeability of 2056 g·m^-2^h^-1^ and a sensitivity of 138.5 kPa^-1^ in the pressure range of 0–30 kPa. Using this sensor in combination with electrocardiogram monitoring, they evaluated systolic blood pressure with an accuracy of 0.6 ± 3.57 mmHg and diastolic blood pressure with an accuracy of 0.7 ± 3.72 mmHg, meeting the criteria set by the Association for the Advancement of Medical Instrumentation.

## 4 Iontronic strain sensor (ISS)

Strain sensors, vital for applications such as body motion tracking, healthcare, rehabilitation, and athletic performance evaluation, need to adhere closely to the skin, be flexible, and be adept at capturing wide-ranging dynamic movements (Souri et al., 2020; Shen et al., 2022). Moreover, they should withstand repeated mechanical stresses to ensure continuous monitoring (Liu et al., 2018). Iontronic materials are distinguished by their exceptional mechanical pliability, featuring significant stretchability (0%–1,000%) and fatigue resistance bolstered by self-healing properties (cycle >1,000) (Amoli et al., 2020). Their consistent ionic conductivity guarantees continuous current flow, even under stretching conditions, ensuring on-going monitoring of changes in strain. This section explores various resistive strain sensors rooted in iontronics.

### 4.1 Principle of resistive ISS: deformation-induced resistive change

Many ISSs measure deformation through resistance variation caused by change of geometric dimensions in length and area (Niu and Liu, 2022). Bai et al. (2021) developed ISS consisting of PAAm-sodium carboxymethyl cellulose (CMC) including Ca^2+^ ion for crosslinking and ionic conductivity. The ISS has stretching capacity of up to 1,480%, an ionic conductivity of 1.4 S·m^-1^, and a gauge factor (GF) ranging from 0.62 to 1.42. Moreover, the sensor maintained its sensitivity even after enduring 200% stretching of 500 repetitions. However, ISSs face various limitations, including a non-linear GF and low sensitivity due to the dependence of resistance change only on the deformation caused by constant conductivity under strain. These limitations have prompted extensive research efforts to overcome these issues.

### 4.2 Performance enhancement of ISS

For ISS to function effectively as a wearable sensor, while ISSs should maintain their elasticities and conductivities under significant deformations, they should exhibit sensitivity to electrical signal variations under minor mechanical deformations. Also, ISSs should sustain performance under continuous, repetitive mechanical changes. Various research approaches, including polymer network modulation, alterations in ISS geometry, microstructure engineering within ITL, and the implementation of a double conductive pathway, are being employed to address these requirements. Table 2 lower section, dedicated to resistive strain, provides a brief summary of the performance of recently developed ISSs through the mentioned approaches.

#### 4.2.1 Polymer network modulation

**FIGURE 6:**
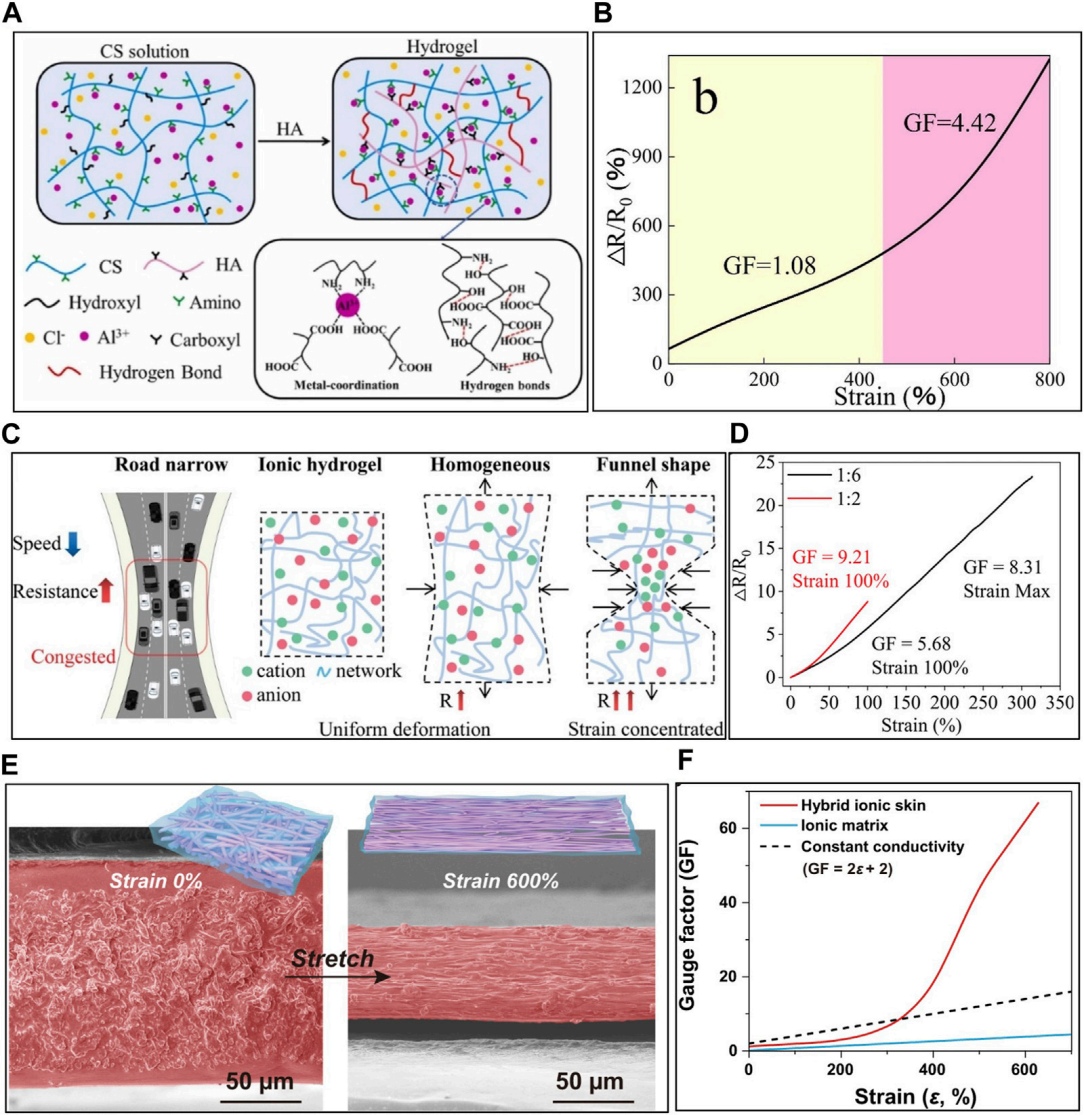
The strategies for the enhancement of sensing performance of ISS. **(A)** Schematic diagram and intermolecular interaction of Chitosan/Hyaluronic acid/Al^3+^ hydrogels, and **(B)** sensitivity of the ISS. **(C)** Ion conduction modulation through funnel shape-induced strain concentration and **(D)** sensitivity of funnel shaped ISS. **(E)** Hybrid ionic skin consisting of elastic nanomesh filled with ionic gel and **(F)** sensitivity of hybrid ionic skin. Reprinted with permission from **(A,B)** Gao et al. (2023), **(C,D)** Zhao et al. (2023a), **(E,F)** Wang et al. (2022b).

Polymer networks have a profound influence on the performance of ISSs, affecting parameters like stretching behavior, electrical conductivity, adhesion, GF, and linearity. Researchers have developed various techniques for polymer network modulation to address these critical aspects. Gao et al. (2023) employed a double network hydrogel by introducing Al^3+^ ions into a combination of chitosan (CS) as a polycation and hyaluronic acid (HA) as a polyanion (Figure 6A). This led to the ionic coordination between hydroxyl and amino groups of CS and carboxyl groups of HA, along with hydrogen bonding between the two polymers. These noncovalent, dynamic, and reversible cross-linking interactions produced a material capable of remarkable stretching (up to 2000%) and exhibited a high self-healing efficiency (97%). This outstanding mechanical property translated into a ISS with a GF of 4.42, an 800% sensing range, and maintained performance even after 300 repetitions at 50% strain (Figure 6B) (Gao et al., 2023). However, such performance improvements are ultimately limited by changes in resistance based on the globally geometric deformation during stretching. This limitation makes it challenging to achieve high sensitivity.

#### 4.2.2 Geometry of ISS

The geometry of the sensor could not only cause a change in resistance based on global deformation but also boost resistance based on extreme and local geometric deformation by hindering ionic flow, similar to the traffic congestion on a narrow road. Zhao et al. (2023a) prepared the funnel-shaped ISS consisting of PVA, Gly, and FeCl_3_ and attaching porous thermoplastic polyurethane (TPU) at both ends (Figures 6C, D). The rigid porous TPU focuses strain on the center of the funnel shape of the sensor during stretching, which reduces ionic flow and improves the relative resistance change. As a result, the GF of the sensor increased to 9.21 at 100% strain, which is 4.6 times higher than the GF of the thin film ISS.

#### 4.2.3 Microstructure engineering

Beyond macro-shapes, researchers have improved ISS performance by modulating the microstructure. Microstructures within ITLs have lower modulus, allowing for more substantial deformation under stress, impacting ionic conductivity and resistance (Chen et al., 2022a; Chen et al., 2022c; Ji et al., 2022). Ou et al. (2022) employed tris(2-methacryloyloxyethyl) borate, 1-[3-(methacryloyloxy)propylsulfonyl]-1(trifluoromethanesulfonyl)imide, polyhedral oligomeric silsesquioxane (POSS), 2-propenoic acid to create a liquid-free ITL. The POSS acted as a crosslinker, forming a compact and strong hybrid crosslinked network, leading to reticular wrinkled microstructures. The wrinkled design of sensor, complemented by microstructures that flexibly expanded and contracted during stretching, resulted in a varied GF. The GF was 1.09 for strains less than 30%, 2.45 for strains between 30% and 60%, and reached 7.03 at 100% strain. The microstructure of the sensor not only facilitates deformation but also induces a change in the shape of ion channels due to stretching, thereby altering ionic conductivity, and enhancing the performance. Wang et al. (2022b) prepared a self-healing hybrid ionic skin with PU nanomesh filled with ionic matrix composed of P(AAm-co-AA), HA, and Ca^2+^ (Figures 6E, F). During stretching, random nanofiber initially aligned, leading to increased ionic conductivity and lower GF due to the lower resistivity at the start of stretching (0%–200%). However, beyond 200%, the aligned PU nanofibers compressed the soft ionic matrix, resulting in denser nanopores and significantly increasing resistivity. This led to a rapid increase in GF during stretching, achieving a record-high GF of 66.8 at 630% strain. This high sensitivity at large strains enabled the sensor to maintain a stable response even after 2,500 repetitions of stretching at 200% strain.

#### 4.2.4 Double conductive pathway

**FIGURE 7:**
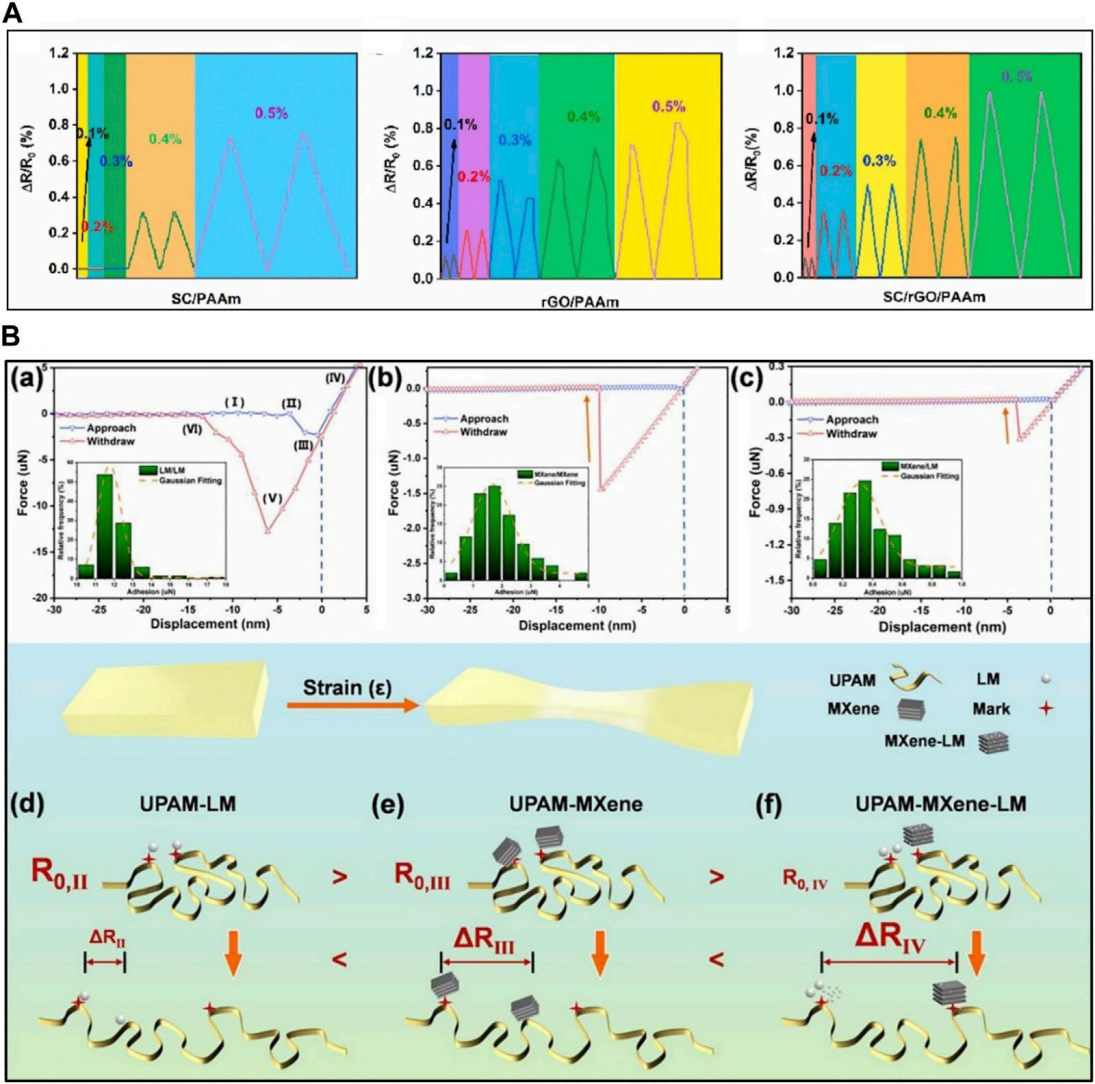
The strategies for the enhancement of sensing performance through dual-conductive pathway. **(A)** The minimum strain of ion-conductive sensor (SC/PAAm), electron-conductive sensor (rGO/PAAm), dual-conductive sensor (SC/rGO/PAAm) during tensile loading and unloading. **(B)** Combination of LM and MXene in ionic gel. Reprinted with permission from **(A)** Wang et al. (2021), **(B)** Dong et al. (2023).

Ionic conductivity provides stable current flow within the ITL during stretching, but sensitivity can be low at small strains since resistivity changes depend entirely on strain variations. In contrast, electrical conductivity relies on percolation-based resistance changes between conductive fillers within the polymer matrix, allowing for resistance changes even at small strains. Combining these two types of conductivity can enhance ISS performance. For example, Wang et al. (2021) incorporated sodium caseinate (SC) for ionic conductivity and reduced graphene oxide (rGO) for electronic conductivity into a PAAm hydrogel, creating a double electrical pathway in ISS. SC/PAAm hydrogel, operating based on ionic conductivity, exhibited no resistance changes within the 0.1%–0.3% strain range but showed stable resistance changes at 0.4%–0.5% due to its steady conductivity (Figure 7A) (Wang et al., 2021). In contrast, rGO/PAAm, relying on electronic conductivity, operated within the small strain range of 0.1%–0.5%, but resistance changes became unstable beyond 0.3% due to the interruption of the conductive pathway. SC/rGO/PAAm showed resistance changes at all small strain levels, and the stability of ionic conductivity enabled stable resistance changes even at 0.3%–0.5% strain. The double conductive pathway in SC/rGO/PAAm resulted in higher GF could improve small strain recognition, recording stability, and sensitivity in ISSs compared to the mono-conductive pathway of the other two ISSs.

Combining solid and liquid conductive fillers can enhance performance. Dong et al. (2023) created an ISS with a double conductive pathway by adding solid-type filler Ti_3_C_2_T_x_ (MXene) and Ga/In (LM) within a PAAm ionic gel based on urea/ChCl deep eutectic solvents (UPAAm-MXene-LM) (Figure 7B). The interface between MXene and LM in UPAAm-MXene-LM exhibited weaker adhesion than MXene-MXene and LM-LM interfaces, allowing for rapid separation under minimal external pulling force, resulting in quick resistance changes. UPAAm-MXene-LM achieved a GF of 7.85 in the 0%–130% strain range, 15.47 in the 130%–240% range, and high sensitivity with a double conductive network.

### 4.3 The application of ISS

**FIGURE 8:**
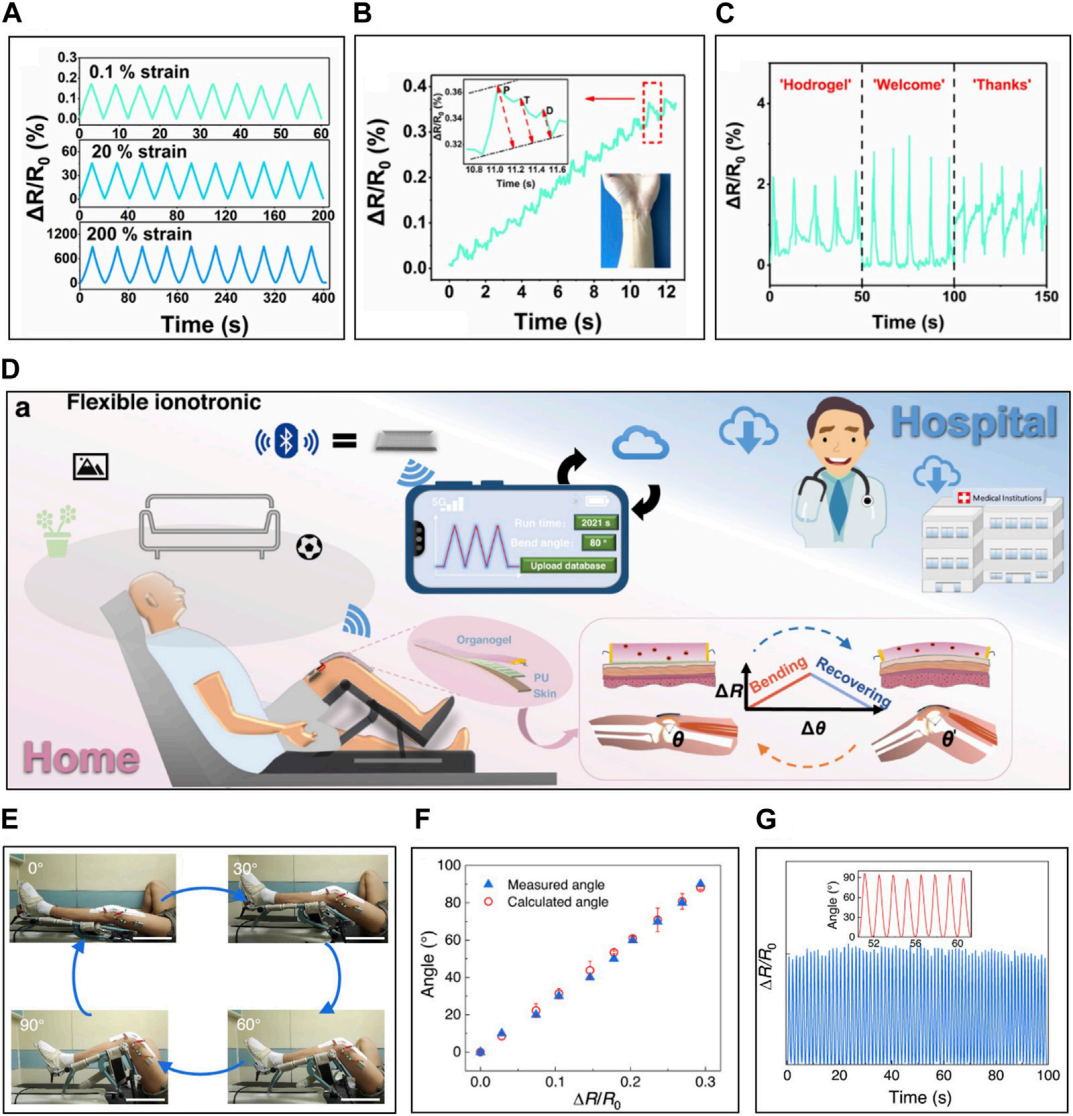
The application of ISS. **(A)** Relative resistance changes of ISS under different tensile strains **(B)** Monitoring of wrist pulse. **(C)** Monitoring of phonation. **(D)** The exercise rehabilitation sensing system with ISS, wireless transmitter, and continuous passive motion equipment for telemedicine. **(F)** Passive bending motion of patient with ISS attached, **(E)** correlation between actual angles and angles calculated from signals measured via ISS, and **(G)** monitoring of angle through ISS during joint movement. Reprinted with permission from **(A–C)** Zhang et al. (2023a), **(D–G)** Tang et al. (2023).

ISSs, leveraging improved sensing performance, are applicable to monitor not only large and slow mechanical deformations such as knee bending and locomotion, but also small and fast mechanical deformations like vocal vibrations and wrist pulse waves. Zhang et al. (2023a) prepared an ISS by incorporating K^+^ and Na^+^ ions into a carboxyethyl chitin/PAAm hydrogel (Figures 8A–C). This sensor achieved a GF of 4.69 within the 0%–300% strain range, and demonstrated response and relaxation times of 61 m and 89 m, enabling the detection of 0.1% strain (Figure 8A). Leveraging these sensing capabilities, the sensor could monitor pulse waveforms on the wrist, distinguishing percussion peaks, tidal peaks, and diastolic peaks (Figure 8B). When placed at the neck position, it monitored the movements of the throat and epidermis and identified specific and repetitive signal patterns associated with speaking certain words, thereby demonstrating its potential for phonetic recognition (Figure 8C).

ISSs, with their excellent performance, are being investigated for their potential use in remote healthcare by combining them with a wireless transmitter (Figures 8D–F) (Tang et al., 2023). Tang et al. (2023) created an ISS by transforming a PVA-PAAm ionic hydrogel with LiCl into an organogel using Gly for solvent exchange. The Gly-based organogel exhibited a wide environmental tolerance of −60°C to 45°C and rapid self-healing capability within 60s. Moreover, it displayed excellent sensing performance with a GF of 1.5 and high linearity (*R* ^2^ = 0.99) over the range of 0%–200%. Leveraging this, the resistivity change ratio showed a high linearity concerning the bending angle, allowing precise detection of bending angles through resistance changes, not merely by bending the sensor. When connected to a wireless transmitter, the ISS system was attached to the knee of patients to send real-time knee bending data to healthcare providers, demonstrating its practical use in rehabilitation therapy.

## 5 Iontronic humidity sensor (IHS)

### 5.1 Principle of resistive and capacitive IHS: absorption-induced conductivity change

Monitoring respiration is essential for diagnosing respiratory diseases such as influenza, asthma, as well as respiration failure conditions like sleep apnea syndrome (SAS) (Li et al., 2022b). Respiration contains a significant amount of moisture, making it possible to monitor respiration by utilizing highly sensitive and fast responsive humidity sensors (Luo et al., 2022a; Zhang et al., 2022b). Furthermore, it’s possible to measure skin moisture conditions by humidity sensor because human skin contains a substantial amount of moisture.

**FIGURE 9:**
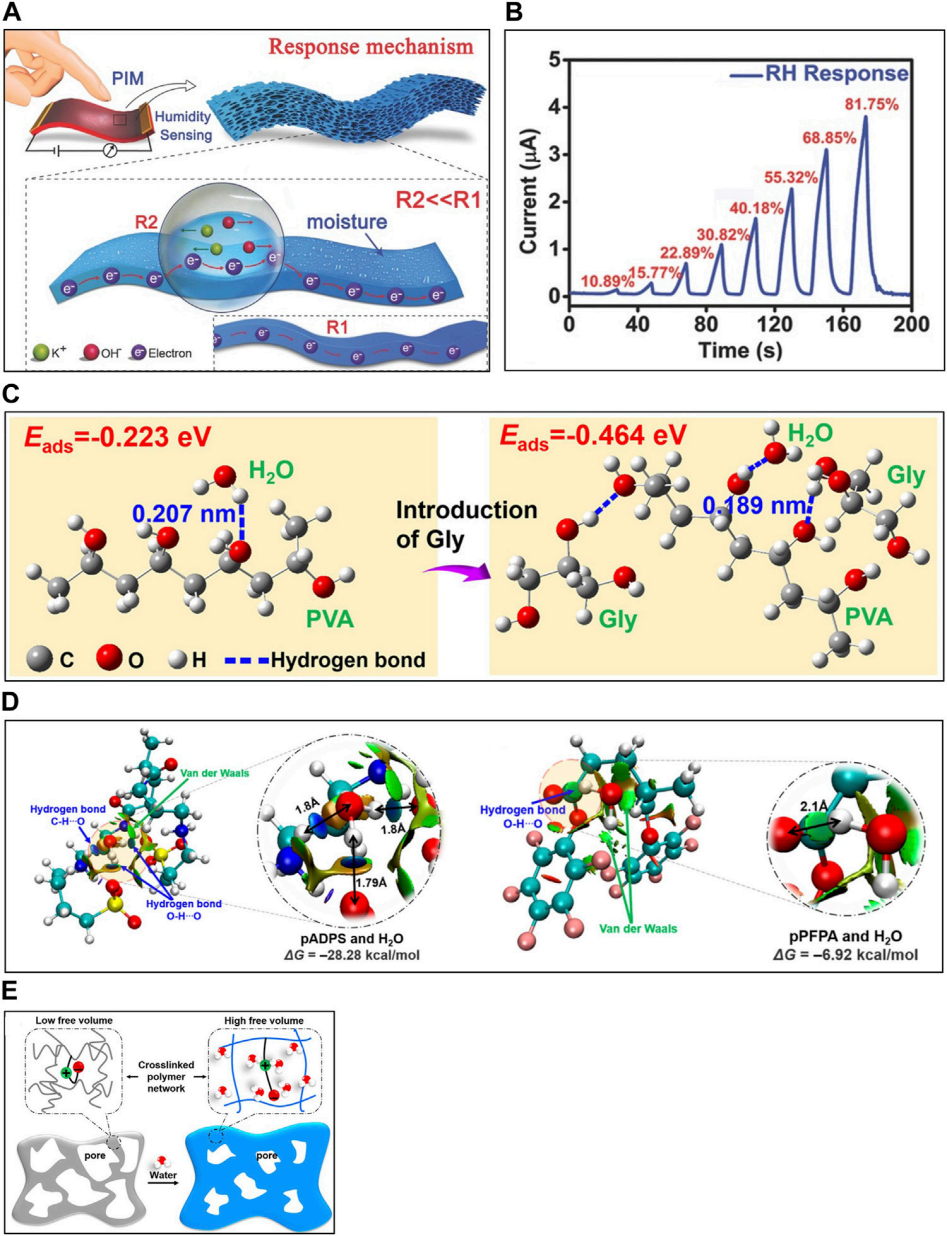
The principle of IHS and the strategies for the enhancement of sensing performance. **(A)** Mechanism of IHSs with electrolytes. **(B)** Current change of PVA matrix-based humidity sensor with KOH electrolytes in response to relative humidity. **(C)** DFT analysis of the adsorption of water molecules in PVA without (left) and with (right) glycerol. **(D)** Structure and binding energy with water molecule of PFPA-branched PEI matrix with (left) and without (right) ADPS zwitterion. **(E)** Illustration of dehydrated and hydrated status of porous structured IHS. Reprinted with permission from **(A,B)** Li et al. (2017b), **(C)** Ding et al. (2023), **(D,E)** Li et al. (2021).

IHSs utilizing hydrophilic polymer matrices operate a formed hydrogen bonding with the water molecule and hydrophilic functional groups such as hydroxyl and carboxylic groups (Li et al., 2017b; Wu et al., 2019). The levels of water absorption in IHS change based on external humidity conditions. Water molecules cause the dissociation of electrolytes such as NaCl or KOH, which increases the ion concentration (Wang et al., 2020a). Water molecules also lead to the unfolding of polymer chains, reduced resistance to ionic movements, and enhanced ionic conductivity (Wu et al., 2019; Wang et al., 2020a). Given that electrolytes dissociate more readily than water molecules protonate, iontronic sensors demonstrate significantly greater sensitivity than their traditional counterparts (Figure 9A) (Li et al., 2017b). For example, Li et al. (2017b). Fabricated a PVA matrix-based IHS with KOH electrolytes, achieving a sensitivity of 9 per root of relative humidity (RH%) (Figure 9B). Wang et al. (2020b) developed IHS with a cellulose matrix and KOH electrolytes, exhibiting a sensitivity of approximately 2.3 within the range of 11.3%–97.3% and a rapid response time of 6s and recovery time of 10.8s.

### 5.2 Performance enhancement of IHS

Efforts are underway to enhance the sensitivity of IHSs by increasing the hydrophilicity of matrix, aiming to boost their water absorption capability. This involves adding Gly or EG as additives(Wu et al., 2019; Ding et al., 2023). Gly easily forms hydrogen bonding with water molecule. Wu et al. (2019) formed an organohydrogel by substituting water with Gly or EG in a hydrophilic PAAm/k-carrageenan polymer matrix. This IHS showed approximately 40 times increase in sensitivity from 0.24 to 10 compared to water solvent. Additionally, Ding et al. (2023) created an organohydrogel in a PVA/cellulose nanofiber (CNF) polymer matrix-based IHS by mixing water and Gly with 1:1 ratio as a solvent. Using density functional theory (DFT) analysis to measure water absorption capability, they found that the energy required for water molecule adsorption onto the matrix decreased from −0.223 eV to −0.464 eV, and the hydrogen bonding length also shortened from 0.207 nm to 0.189 nm when using the hydrophilic Gly (Figure 9C).

Zwitterionic polymer matrix-based IHS achieve high sensitivity due to the dense of cations and anions that interact through dipole interactions with water molecules (Shen et al., 2021). Yang et al. (2023c) developed IHS based on a polymer matrix consisting of polycondensated squaric acid and tris(4-aminophenyl)amine (TAPA). This sensor exhibited a conductivity change approximately 10^4^ times greater at 98% compared to 11% humidity and had a fast response and recovery time of 1 s and 7 s, respectively. Li et al. (2021) created an IHS with a zwitterionic polymer matrix by substituting 3-((3-aminopropyl)-dimethylammonio)propane-1-sulfonate (ADPS) zwitterion for pentafluorophenyl acrylate (PFPA)-branched polyethyleneimine (PEI). Through noncovalent interaction and DFT analyses, it was determined that zwitterions establish more strong hydrogen bondings with water molecules than PFPA branched polymers (Figure 9D). The zwitterion-based IHS showcased a remarkable sensitivity of about 600 and a fast response rate of 24 s^-1^.

Enhancing hydrophilicity can boost sensitivity and response speed by facilitating the attraction of water molecules. However, it may also complicate the dehydration process, which decrease the recovery rate. As a solution, developments into the use of porous structures aim to expand the surface area and simultaneously improve both response and recovery rates by shortening the diffusion length. Li et al. (2021) formed porous IHS by blending zwitterionic polymer matrix with poly(methyl methacrylate) (PMMA) and then created the porous structure through selective etching with chloroform (Figure 9E). Under the humidity conditions change with 50%–90%, the saturated capacitance increased from 1,200 nF to 1700 nF, and the response and recovery times decreased from 56 s to 10.2 s and 154 s to 10.2 s, respectively.

### 5.3 Applications of IHS

**FIGURE 10:**
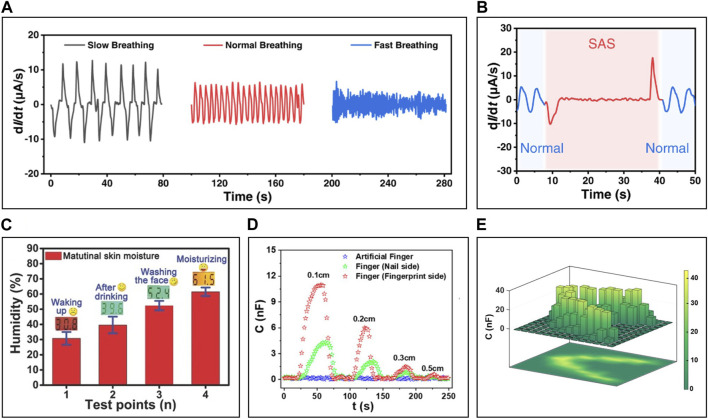
The applications of skin-interfaced IHS. **(A)** Signals of breathing at different frequencies and **(B)** patients with SAS detected by silk fibroin polymer matrix-based humidity sensor with LiBr electrolytes. **(C)** Comparison of PVA matrix-based humidity sensor with KOH electrolytes and commercial humidity sensor. **(D)** Capacitance change upon assessments of fingers and **(E)** perceiving finger motion of V-shaped path to the humidity sensor based on ADPS zwitterion with PFPA-branched PEI matrix at different distances. Reprinted with permission from **(A,B)** Li et al. (2022b), **(C)** Li et al. (2017b), **(D,E)** Li et al. (2021).

IHSs with such high sensitivity and response rates are being utilized to monitor human respiration (Li et al., 2017b; Li et al., 2022b). Li et al. (2022b) developed IHS capable of diagnosing SAS by assessing the intensity, frequency, and rhythm of respiration. This IHS is crafted from an ionic gel, which comprises a silk fibroin polymer matrix, graphene oxide to enhance hydrophilicity, and LiBr electrolytes. It can accurately monitor rapid respiratory rates of up to 60 breaths per minute with a response time of approximately 1s, facilitating a clear distinction between SAS and normal breathing patterns (Figures 10A, B).

Li et al. (2017b) utilized IHS, based on a PVA matrix containing potassium hydroxide (KOH), to evaluate skin conditions by measuring its water content. The sensor gauged the water content of skin following events such as waking up, drinking, face-washing and moisturizer application. The recorded measurements aligned consistently with values from a commercial water measurement instrument (Figure 10C).

Employing a highly sensitive IHS allows for the detection of finger assessments without direct contact. It measures shifts in ambient humidity resulting from the moisture present on human skin. Li et al. (2021) achieved this assessment of fingers from distances between 0.1 cm and 0.5 cm to the sensor, with intervals of 0.1 cm. The sensor could distinguish between sources with different intrinsic water content, such as the nail side and a dry artificial finger, as these objects approached (Figure 10D). Additionally, with a rapid response rate and remarkable sensitivity—24 s^-1^ and 600, respectively—the sensor precisely detected ‘V’ shaped finger movements within a 13 × 13 matrix (Figure 10E).

In respiration monitoring, it is also possible to distinguish normal and abnormal respiration based on frequency with high sensitivity. However, accurately monitoring the intensity and rhythms of fast respiration with tens Hz using a humidity ionic sensor that has a response time in the order of seconds is limited. This limitation arises due to the long diffusion length caused by absorption in the bulk structure, so the research is necessary to reduce diffusion length.

## 6 Ionic temperature sensor (ITS)

### 6.1 Principle of resistive or capacitive ITS: temperature-induced ion mobility shift

The human body typically maintains temperature of around 36.5°C, but deviations can result from various conditions, including bacterial infections, inflammation, cardiovascular, and pulmonary diseases (Li et al., 2017a; Xu et al., 2020). Consequently, skin-interfaced temperature sensors are crucial for diagnosing and managing these conditions by tracking body temperature (Trung et al., 2018; Lou et al., 2020). Developments in skin-interfaced temperature sensors are paving the way for artificial skin aiming to emulate thermoreceptors of human skin, with potential applications in prosthetic hands and soft robotics (Hong et al., 2016; Bae et al., 2018).

**FIGURE 11:**
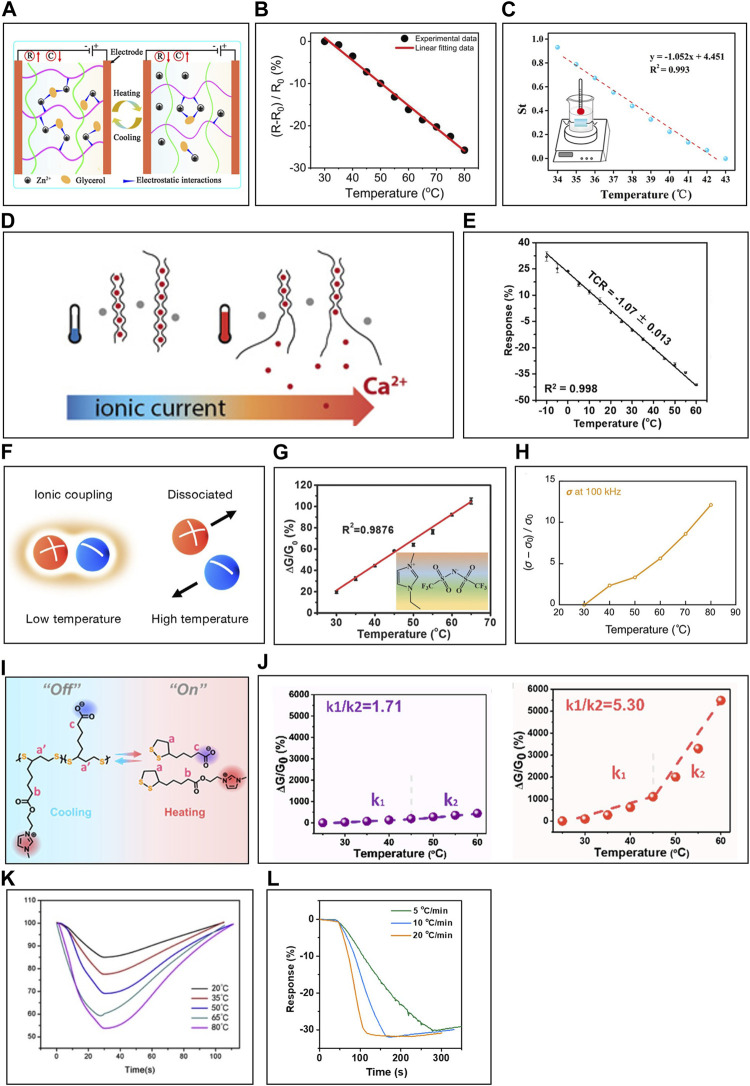
The principle of ITS and the strategies for the enhancement of sensing performance. **(A)** Mechanism of ITS. **(B)** Resistance change of PVA polymer matrix-based temperature sensor with AlCl_3_ at temperature change. **(C)** Resistance change of gelatin/PAAm-clay matrix-based temperature sensor with ZnCl_2_ at temperature change. **(D)** Mechanism of enhanced sensitivity of ionic crosslinked polymer-based temperature sensor. **(E)** Resistance change of Fe^2+^ crosslinked k-carrageenan/PAAm at temperature change. **(F)** Illustration of IL dissociation at low and high temperature. **(G)** Conductance change of PU fiber-based temperature sensor with [EMIM][TFSI] at temperature change. **(H)** Conductivity change of PVA-based temperature sensor with [MTEOA][MeOSO_3_] at temperature change. **(I)** Illustration of mechanism of thioctic acid branching IL polymerization-depolymerization transition. **(J)** Conductance change of [EMIM][HA] (left) and thioctic acid branching IL (right) at temperature change. **(K)** Response/recovery properties of PAA/PAAm-based temperature sensor with CNT. (L) Response properties of CA/PAAm-based temperature sensor with MXene at various temperature change rate. Reprinted with permission from **(A)** Zhu et al. (2020), **(B)** Wen et al. (2021), **(C)** Zhu et al. (2020), **(D)** Di Giacomo et al. (2017), **(E)** Wang et al. (2023c), **(F)** Yamada and Toshiyoshi (2020), **(G)** Gui et al. (2017), **(H)** Yamada and Toshiyoshi (2020), **(I,J)** Chen et al. (2022b), **(K)** An et al. (2020), **(L)** Wang et al. (2023c).

Gel-type ITSs detect changes in ionic conductivity, reflecting ion mobility shifts within the solvent due to temperature fluctuations (Wu et al., 2020; Wen et al., 2021). As the temperature rises, metal cations attached to the polymer matrix through electrostatic interactions often transition to free ions (Figure 11A) (Zhu et al., 2020). These internal free ions’ mobility is influenced by electromigration due to an external electric field, and their diffusivity depends on temperature following the Arrhenius equation. Wen et al. (2021) designed an organohydrogel ITS using a PVA polymer matrix and AlCl_3_ electrolyte. It showcased remarkable mechanical attributes, enduring up to a 480% ultimate tensile strain and exhibiting a toughness of 937.403 kJ ⋅ m^-3^. Its conductivity was altered by 0.536% for every 1°C change (Figure 11B). In a related endeavor, Zhu et al. (2020) introduced an organohydrogel ITS, crafted from a gelatin/PAAm-clay composite and infused with ZnCl_2_ electrolytes. This sensor achieved over 500% stretchability and registered a 1.052% shift in conductivity for each 1°C change (Figure 11C). However, ionic conductivity in ionogels lags behind the electrical conductivity observed in traditional TCR-based thermistors. Therefore, strategies are needed to amplify sensitivity for precise readings of slight body temperature fluctuations.

### 6.2 Performance enhancement of ITS

#### 6.2.1 Utilizing ionic crosslinked polymer matrix

Polymers like alginate and carrageenan, which have functional groups with negative charges, establish strong electrostatic bonds with metal cations carrying 2^+^ or 3^+^ charges, leading to crosslinking. As the temperature rises, these electrostatic bonds reduce in strength, increasing the mobility of the polymer chains and facilitating the dissociation of the ionic crosslinks. Consequently, metal cations are freed more readily, resulting in a higher ion concentration (Figure 11D) (Di Giacomo et al., 2017). ITSs, based on ionic crosslinked polymer matrices, display enhanced sensitivity with rising temperatures due to simultaneous increases in ion mobility and concentration. Di Giacomo et al. (2017) devised such a sensor using pectin, ionic crosslinked via Ca^2+^ ions, operating under 0.1 V between 10°C–55°C. Similarly, Wang et al. (2023c) designed a ITS by integrating k-carrageenan ionic crosslinked with Fe^2+^ ions into PAAm. This device showed a 1.07% shift in conductivity for each 1°C change between -10°C – 60°C (Figure 11E).

Despite advancements, challenges still remain in amplifying sensitivity because of the constrained solubility of electrolytes. Moreover, only a small fraction of cations in the polymer matrix engage in ionic crosslinking. To address this, IL are being considered as potential electrolytes with their high ionic conductivity (Shiddiky and Torriero, 2011).

#### 6.2.2 Utilizing IL electrolyte

IL are salts made up solely of cations and anions with liquid phase at room temperature. As the temperature rises, the binding forces between these ions decrease, which increases the probability of ion dissociation (Figure 11F) (Yamada and Toshiyoshi, 2020). This decreases the IL’s viscosity which is influenced by the forces between the cations and anions (Ota et al., 2014). So, ionic mobility could be enhances not just from increased diffusivity but also from reduced viscosity (Vila et al., 2006; Ganbold et al., 2015). Empirically, the temperature-dependent conductivity of IL follows the Vogel-Tamman-Fulcher equation (Vila et al., 2006; Stoppa et al., 2010). Gui et al. (2017) fabricated an ITS by soaking polyurethane (PU) fiber in EMIM][TFSI]). It exhibited a 2.38% conductivity change per 1°C within the range of 30°C–65°C (Figure 11G). Yamada and Toshiyoshi (2020) achieved approximately a 24% conductivity change per 1°C within 30°C–80°C range by using a PVA with tris(2-hydroxyethyl)methyl ammonium ethylsulfate ([MTEOA][MeOSO_3_]) (Figure 11H). Chen et al. (2022b) further enhanced temperature-induced viscosity changes by introducing thioctic acid branching to the IL. It allowed a reversible shift between the states of polymerized oligomer and free monomer (Figure 11I). This ITS showcased a substantial conductivity alteration of 156.79% per 1°C when temperatures exceeded 45°C, attributed to its polymerization-depolymerization transition. Notably, this sensitivity surpassed that of the conventional IL [EMIM] hexanoic acid (Figure 11J).

#### 6.2.3 Additive with high thermal conductivity

In healthcare monitoring, a response rate of temperature sensor is essential for rapidly diagnosing temperature fluctuations. Ionic gels have lower thermal conductivity due to low thermal conductive polymers and solvents (Wang et al., 2023c). To improve it, researchers have incorporated additives like carbon nanotubes or MXene, known for their high thermal conductivity (Kumanek and Janas, 2019; Yi et al., 2022). For instance, An et al. (2020) integrated CNT into PAAm/PAA hydrogel, consistently detecting temperature variations from 20°C–80°C (Figure 11K). Similarly, Wang et al. (2023c) employed MXene to enhance the thermal conductivity of ITS, enabling accurate tracking of temperature variations within 20°C–40°C (Figure 11L).

### 6.3 Applications of ITS

**FIGURE 12:**
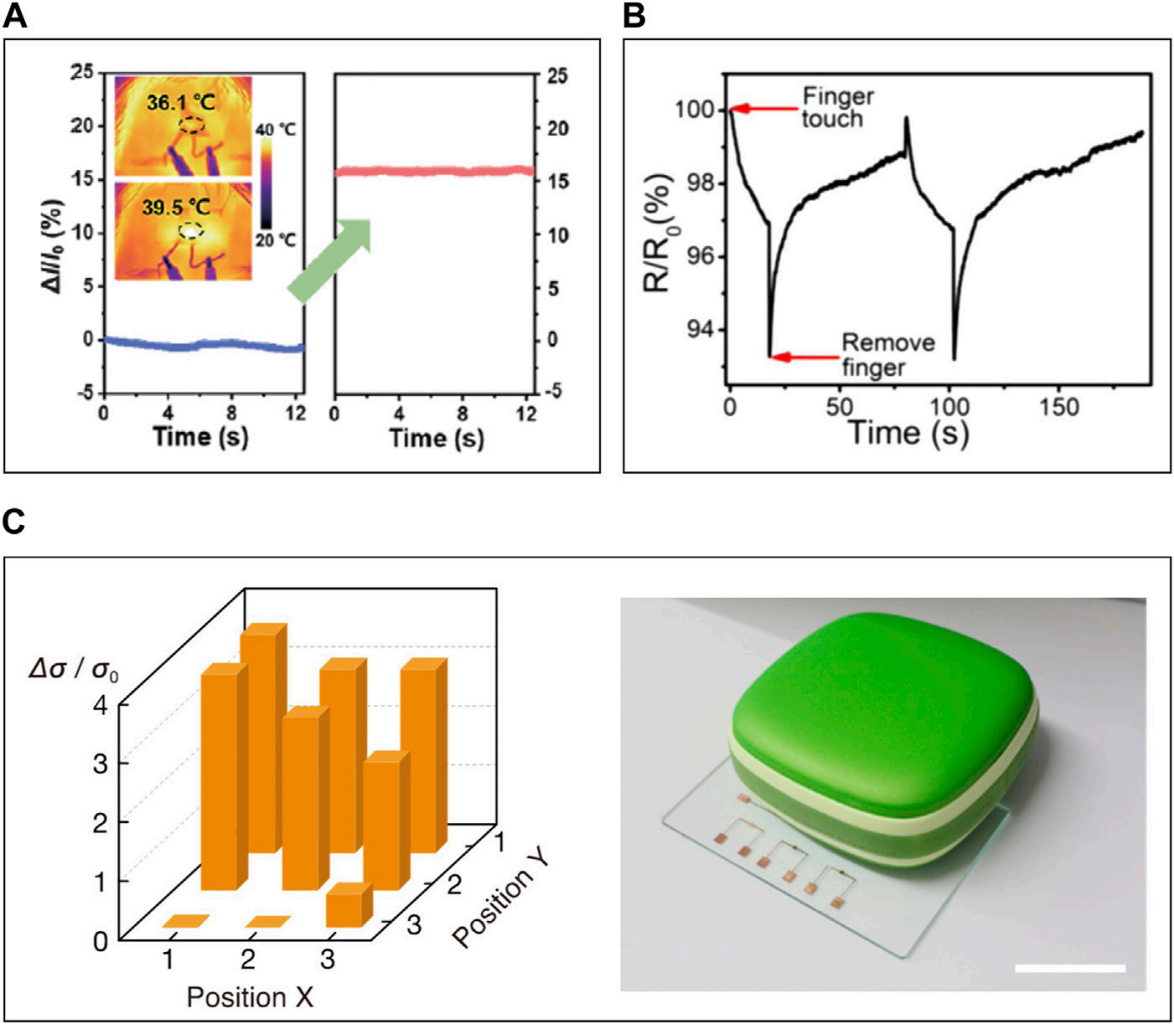
The applications of skin-interfaced ITS. **(A)** Current change of PAA/cellulose nanocrystal-based ITS on forehead with temperature change. **(B)** Resistance changes of PAAm/k-carrageenan-based ITS with attachment/detachment of finger. **(C)** Conductivity change of PVA with [MTEOA]^+^[MeOSO_3_]^−^ ITS array in response to placement of hand warmer. Reprinted with permission from **(A)** Lu et al. (2022), **(B)** Wu et al. (2018), **(C)** Yamada and Toshiyoshi (2020).

Researches have been conducted to detect human body temperature change using ITSs with such high sensitivity and response rate. Lu et al. (2022) detected human fever using a PAA/cellulose nanocrystal polymer matrix-based ITS. They confirmed temperature changes from 36.1°C to 39.5°C, simulating artificial fever on skin, with a 16% current change (Figure 12A). Highly sensitive ITSs also can be used to detect finger touch with elevated temperatures at room temperature. Wu et al. (2018) measured finger touch and release through a 7% resistance change utilizing a PAAm/k-carrageenan polymer matrix-based hydrogel with KCl electrolytes (Figure 12B). Moreover, Yamada and Toshiyoshi (2020) fabricated an array of ITSs, enabling the detection of resistance changes for each sensor and determining the position of the contacting object (Figure 12C). This demonstrates the potential for development towards artificial skin that mimics the thermoreceptors in the skin. ITS holds promise for healthcare monitoring and artificial skin applications, but practical implementation still faces hurdles. A key challenge is the requirement for increasing sensitivity. While current ITSs typically offer a resolution between 0.1 and 1°C—suitable for everyday health monitoring—medical applications often demand precision up to 0.01°C (Takei et al., 2015). Additionally, accurately detecting core body temperature via skin readings can be a problem as skin temperature is significantly affected by external conditions. Thus, strategies to insulate these sensors from environmental heat variations are necessary (Xu et al., 2020).

## 7 Multi-modal sensors

Combining multiple sensors in one device enhances data collection and analysis by optimizing resource usage, reducing redundancy, and improving efficiency. This integration of diverse sensing modalities enables cost-effective applications in various fields like decision-making, real-time monitoring, healthcare, smart environments, and industrial automation. Recent efforts have focused on developing multi-modal sensing devices, including iontophoretic sensors, using various techniques to improve sensitivity and modulate external components. The following examples explore different methods for integrating a growing range of modalities.

### 7.1 Bi-modal sensors

#### 7.1.1 Pressure-temperature

**FIGURE 13:**
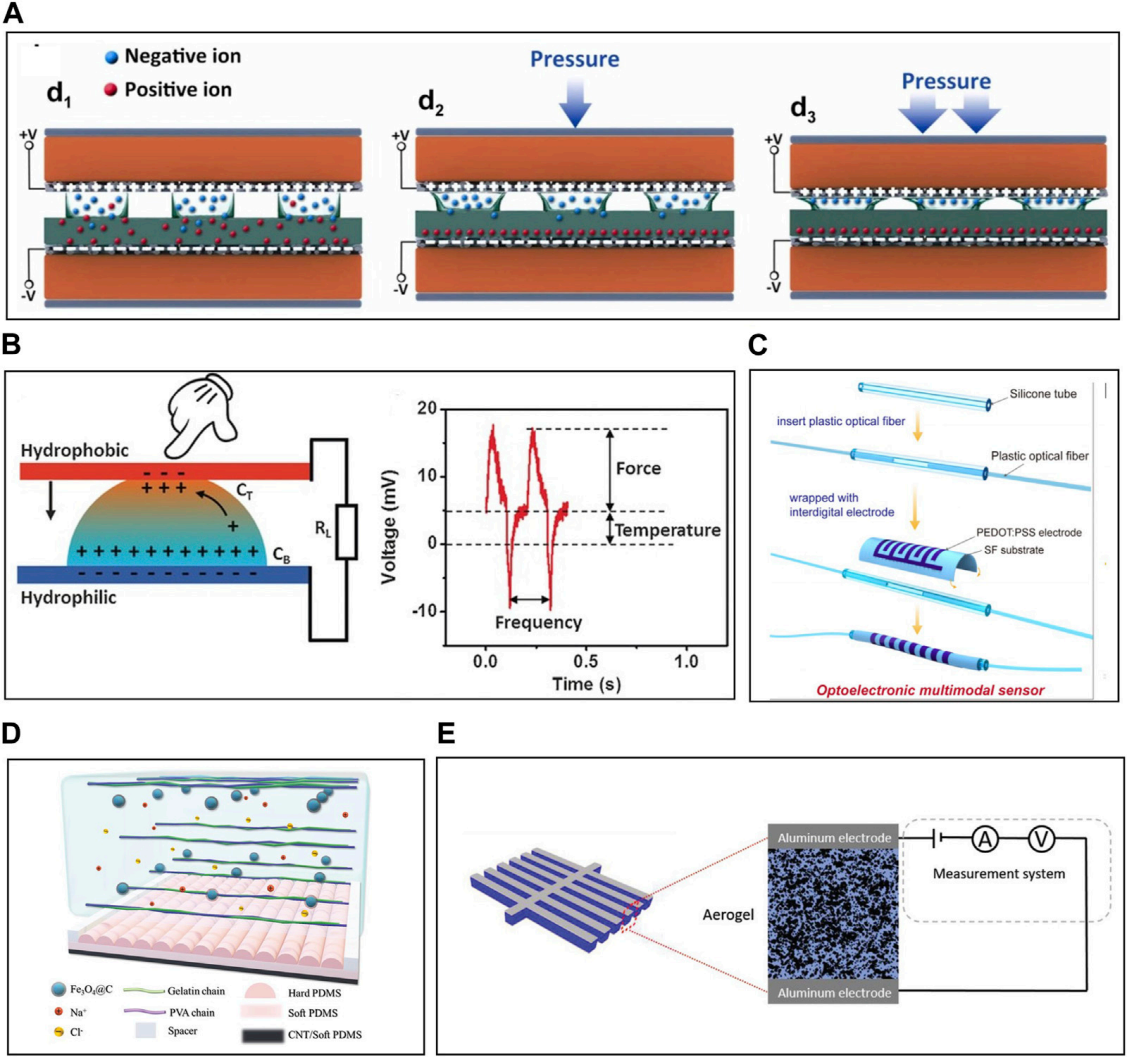
The principle of multi-modal sensing. Schematics of multi-modal sensing strategy for **(A)** Pressure-Temperature **(B)** Force-Temperature, **(C)** Pressure-Temperature-Proximity **(D)** Pressure-Temperature-Strain, **(E)** Pressure-Temperature-Humidity. Reprinted with permission from **(A)** Li et al. (2023), **(B)** Liu et al. (2016), **(C)** Wang et al. (2023a), **(D)** Zhang et al. (2022a), **(E)** Han et al. (2019).

Most ionic conductors exhibit the capability to sense both pressure and temperature by leveraging the EDL associated with pressure-dependent interfacial capacitance and temperature-dependent dielectric constants (Li et al., 2023). As shown in Figure 13A, this can be structured in a capacitive electrode/ionic conductor/electrode configuration (Li et al., 2023). In a specific instance, Li et al. (2023) fabricated a composite-based single cup-shaped microwell array structure. This structure incorporated a layer of TPU and an IL [EMIM][TFSI], situated between silver nanowire (AgNW)/PDMS based electrodes. This design led to a pressure sensitivity of 87.75 kPa^-1^ and an impressive resolution of 0.22 Pa, attributed to the EDL between the electrode and the ITL. The temperature sensitivity was 52.77%·°C^−1^, with a remarkable temperature resolution of 0.1°C. Overall, the sensor could measure pressures up to 170 kPa and temperatures between 40°C and 90°C.

#### 7.1.2 Force-temperature

Based on the phenomena of reverse electrowetting observed in IL droplets, Liu et al. (2016) could measure alternating pulse voltage which correlates with applied forces. Also, direct voltage measured from phenomena of thermo-galvanic effects can quantify temperature gradient across it (Figure 13B) (Liu et al., 2016). In a specific configuration, the sensor comprises a hydrophilic ITO electrode/K_3_[Fe(CN)_6_]/K_4_[Fe(CN)_6_] 0.01 M droplet/hydrophobic ITO electrode, forming an EDL capacitor at the electrode interface where charge transfer occurs in response to external stimuli. Measurement of voltage across the temperature difference between the two flat plates revealed a temperature sensitivity of −1 mV·K^−1^ with a resolution of approximately 0.1 K (Liu et al., 2016). When a periodic force was applied to the flat plate, a 5 μL droplet with a deformation of 0.76 mm and a frequency of 4 Hz generated a peak voltage of +5.6 mV.

To isolate and evaluate the output voltage (U) in an environment where temperature and force are intertwined, a temperature coefficient, B(T), was introduced to partition the internal variables of the function into the voltage (U_B_) attributable to the thermogalvanic effect and the voltage (U_P_) induced by the deformation amplitude and frequency. This relationship can be expressed as U = U_B_ + U_P_B(T) (Liu et al., 2016). Analytical examination confirmed that B(T) displays a linear relationship with temperature change, validating the possibility to independently obtain U_B_ (Liu et al., 2016).

#### 7.1.3 Strain-humidity

When a polyelectrolyte bilayer, with one layer positively and the other negatively charged, is assembled, it possesses the capability to rectify an external voltage based on the migration of specific ions (e.g., H^+^, OH^−^) (Ying et al., 2020). Application of compressive strain to thin the bilayer thickness induces migration of counter ions, and moisture absorption levels alter ion concentrations(Ying et al., 2020). Based on those phenomena, as an example, a bilayer double-network hydrogel was utilized as the sensing material (Ying et al., 2020). The matrix comprised agarose-PAAm (Agar-PAAm), composed of poly(diallyl dimethylammonium chloride) (PDAC) as a positively charged polyelectrolyte, and poly(sodium 4-styrenesulfonate) (PSS) as a negatively charged polyelectrolyte (Ying et al., 2020). Additionally, the hygroscopic material, EG, was incorporated within the matrix to enhance ambient stability (Ying et al., 2020). The introduction of 50% (v/v) EG within the bilayer was shown to sustain current density and rectification ability even after 3 days in an environment at 21°C and relative humidity (RH) of 60%–65% (Ying et al., 2020). Regarding mass changes, the changing addition of EG maintained approximately RH of 67%–73% of the original mass at RH 56%, and around 50% of the original mass even in a low RH environment of 13% (Ying et al., 2020).

To assess strain sensing capabilities of this bilayer, four physical variables—resistance, capacitance, open circuit voltage (OCV), and short circuit current (SCC)—were measured, with a focus on resistance and open circuit voltage (Ying et al., 2020). Under the application of an external voltage of 3 V, resistance at 65% RH reduced from 20.23 kΩ to 4.54 kΩ with a change in compressive strain from 10% to 50% (Ying et al., 2020). The built-in potential, formed by mobile ions in the depletion zone between the bilayers, changes with deformation and can be measured in a pulsed form (Ying et al., 2020). With compressive strain changes from 10% to 50%, the self-generated peak of OCV output increased from 1.1 mV to 4.1 mV (Ying et al., 2020). Importantly, this behavior remained durable for up to 800 cycles of repeated compressive loading-unloading cycles (Ying et al., 2020). Regarding humidity changes, the resistance decreased from 327 kΩ to 23.9 kΩ as RH varied from 13% to 85% at 0% strain (Ying et al., 2020).

### 7.2 Tri-modal sensors

#### 7.2.1 Pressure-temperature-proximity

For precise measurement of multiple signals, preserving the independence of physical variables is essential. Achieving this involves the use of ionic conductors, in addition to employing geometric structures and external devices such as light. For instance, an optoelectronic multi-modal sensor has been developed to measure proximity via changes in capacitance dependent on the distance from the sensor, pressure through alterations in light intensity, and temperature through resistance change (Wang et al., 2023a). In this design, an optical fiber was positioned at both ends of a silicone tube, creating a space between the fibers (Wang et al., 2023a). An electrode-based capacitor interdigitated with PEDOT:PSS was fabricated on silk fibroin and wrapped around the silicone tube (Figure 13C) (Wang et al., 2023a).

In the context of proximity measurement, it was observed that the change in capacitance increases with proximity and can be measured up to a distance of 225 mm (Wang et al., 2023a). Targets approaching at different speeds (1.7 cm·s^-1^, 12.8 cm·s^-1^) could be distinguished based on the difference in the slope of the change (Wang et al., 2023a). Notably, there was no significant change in capacitance observed when the temperature ranged from 30°C to 75°C, confirming the absence of interference (Wang et al., 2023a). For pressure measurement, sensitivity was defined as the relative change in output light intensity divided by the change in pressure (Wang et al., 2023a). The sensitivity was calculated as S = 0.42 N^−1^ for the pressure range of 0–1.3 N and S = 0.26 N^−1^ for the range of 1.3 N–2.3 N (Wang et al., 2023a). In the case of temperature, the temperature coefficient of resistance (TCR) was defined as the relative change in resistance divided by the change in temperature (Wang et al., 2023a). Within the detection range of 50°C, TCR was determined to be 7%·°C^−1^, with a resolution of 0.05°C (Wang et al., 2023a).

#### 7.2.2 Pressure-temperature-strain

In this case, it has been measured through changes in color due to strain, changes in resistance of ions due to temperature, and changes in color and triboelectric voltage due to pressure (Zhang et al., 2022a). As shown in Figure 13D, the chromotropic ionic hydrogel is structured in a form containing an aligned polymer chain and a photonic crystal particle array (Zhang et al., 2022a). The core(Fe_3_O_4_)-shell(C) shaped nanoparticles with a diameter of ∼130 nm were synthesized by applying a magnetic field to double network amine/amide abundant gelatin and PVA, followed by freeze-drying with the addition of crosslinking agent (glutaraldehyde) and NaCl while maintaining the field. In addition, the chromotropic hydrogel was fabricated as a structure that can utilize triboelectricity by interfacing wrinkle patterned hard PDMS (modulus ∼2.44 MPa) with 250 μm amplitude as a top electrode, soft PDMS (modulus ∼0.312 MPa) as a bottom electrode, and carbon nanotube/PDMS as a bottom electrode (Figure 13D) (Zhang et al., 2022a).

Strain could be measured by the change of the reflective wavelength based on the Bragg condition, which the wavelength changed from 680 nm to 430 nm when the strain changed from 0% to 110%, and the change rate was −23 nm/% (Zhang et al., 2022a). For temperature sensing, the resistance changed exponentially from 221.5 kΩ to 16.5 kΩ when the temperature changed from 0°C to 50°C, and the TCR of 20.44%·°C^−1^ in the 0°C–20°C range and TCR of 2.54%·°C^−1^ in the 20°C–50°C range (Zhang et al., 2022a). As the concentration of ions increased, the TCR tended to decrease in all temperature ranges, and it can be inferred that elevated initial impedances resulting from lower NaCl concentrations are favored in the context of highly responsive temperature sensors due to the more substantial reductions in resistance as the temperature increases (Zhang et al., 2022a). Also, the alignment by directional freezing plays a role in increasing the TCR by facilitating the movement of ions compared to random or perpendicular networks (Zhang et al., 2022a). In addition, to reduce interference with strain-related signals, the GF of the strain sensor according to the ion concentration was measured, and found that the GF did not change above 0.2 M, so as to balance high TCR and secure strain insensitivity, ion concentration 0.2 M was used (Zhang et al., 2022a). For pressure sensitivity, triboelectric voltage was measured which is generated when the hydrogel and wrinkled PDMS are pressed and released (Zhang et al., 2022a). The pressure was applied from 0 kPa to 9 kPa, and the sensitivity was −528.0 V·kPa^-1^ in the 0 kPa–2 kPa range and −63.7 V·kPa^-1^ in the 2 kPa–9 kPa range, with a response time of 44 m and a recovery time of 133 m (Zhang et al., 2022a).

#### 7.2.3 Pressure-temperature-humidity

There are examples of independent measurements that do not interfere with each other by using resistance change for pressure, steady thermovoltage for temperature, and thermovoltage peak for humidity (Han et al., 2019). Mixed ionic electronic aerogel was prepared through freeze-drying a blend of PEDOT:PSS, nanofibrillated cellulose (NFC), and a crosslinking agent (glycidoxypropyl trimethoxysilane, GOPS) (Han et al., 2019). The respective roles of each component are as follows: PEDOT:PSS contributes to electrical conductivity and the electronic Seebeck coefficient, NFC enhances mechanical strength, and GOPS provides water stability and elasticity (Han et al., 2019). The aerogel is featured at the core and aluminum electrodes positioned at both ends (Figure 13E) (Han et al., 2019).

For pressure measurement, the resistance altered from 68 Ω to 26 Ω due to deformation as the pressure changed from 0 Pa to 300 Pa (Han et al., 2019). For temperature measurement, electronic thermovoltage (V_e_) was employed, and measurements were preferably conducted at RH less than 50% to avoid considering ionic thermovoltage, given the challenges related to ion migration (Han et al., 2019). V_e_ was calculated using the formula V_e_ = S_e_ΔT, where S_e_ represents the electronic Seebeck coefficient and ΔT denotes the temperature gradient (Han et al., 2019). V_e_ exhibited a linear relationship with temperature, with V_e_ ranging from 0 μV to 400 μV as ΔT changed from 0 K to 20 K (Han et al., 2019). Exposure of the aerogel to DMSO vapor, a known secondary dopant in PEDOT:PSS, confirmed that the resistance remained unaffected by the change in ΔT, effectively decoupling pressure and temperature (Han et al., 2019).

Concerning humidity, when the RH exceeded 60%, ion movement needed to be considered, necessitating the calculation of ionic thermovoltage (Han et al., 2019). This was achieved by subtracting V_e_ from V_peak_, representing the shifted maximum voltage in the I-V curve. V_e_ was measured as the shifted voltage at equilibrium after a 20-min period (Han et al., 2019). Additionally, the ionic contribution to the Seebeck coefficient (S_i_) remained constant even with an increase in pressure up to 300 Pa (Han et al., 2019). S_i_ was measured as 620 μV·K^−1^ at RH 60% and 8,100 μV·K^−1^ at RH 90%, affirming the absence of interference between the three variables under specific conditions (Han et al., 2019).

## 8 Conclusion

### 8.1 Summary

In this review, we have highlighted the advanced technology and diverse applications of skin-interfaced iontronic sensors for healthcare monitoring and electronic skin. First, iontronic sensors have been engineered with high flexibility and stretchability in their polymer matrix to operate effectively on skin with severe mechanical deformation. Materials and structural developments on iontronic sensors have enhanced sensitivity through conformal contact which allows the measurement of subtle changes, and self-healability has been incorporated to improve reliability and durability. Next, iontronic sensors with excellent mechanical properties have been investigated for response to various stimuli such as pressure, strain, humidity, and temperature by elucidating mechanisms governing these responses. Multiple stimuli can be independently measured within a single component by utilizing a variety of sensing parameters for different stimuli, which increases device integration density. Lastly, these sensors have been attached to the skin, enabling the precise measurement of various stimuli originating from the skin. This allows for the detection of abnormal body conditions, activities, and more, and the array format enables stimuli mapping.

### 8.2 Outlook

However, extensive further research is imperative to comprehensively assess the long-term stability of ionic conductors for practical applications. The issue of dehydration is of paramount concern, as it significantly impedes the consistent and reliable transmission of signals through these conductors over time. To mitigate this challenge, the utilization of hygroscopic materials or ionic liquids can be considered, but it is evident that a wider array of material-based solutions is necessary. Also, the field necessitates ongoing advancements in both materials and analytical methodologies to enhance electrochemical stability. In the context of dual ion conduction, the accumulation of ions on one electrode leading to concentration polarization is a notable concern, demanding a higher voltage input to maintain the same current output. Additionally, the response time of ionic conductors falls short of that of conventional metal electrodes, underscoring the pressing need for research aimed at augmenting the mobility of internal carriers. Moreover, when applied in healthcare platforms, it is crucial to develop strategies that enhance breathability when these materials are affixed to biological tissues, as the retention of biofluids on tissue surfaces can potentially trigger inflammation. Current approaches involve creating a structural mesh to facilitate the passage of biofluids, but this method has inherent design limitations and manufacturing complexities. As such, materials for healthcare applications must either possess inherent breathability or incorporate channels to facilitate the smooth permeation of biofluids.
